# Genome-wide association studies and modeling of stomatal gas conductance reveal genetic control of water-use efficiency in sorghum

**DOI:** 10.1093/plphys/kiag064

**Published:** 2026-02-16

**Authors:** Anuradha Singh, Linsey Newton, Addie M Thompson

**Affiliations:** Department of Plant, Soil and Microbial Science, Michigan State University, East Lansing, MI 48824, United States; Plant Resilience Institute, Michigan State University, East Lansing, MI 48824, United States; Department of Plant, Soil and Microbial Science, Michigan State University, East Lansing, MI 48824, United States; Department of Plant, Soil and Microbial Science, Michigan State University, East Lansing, MI 48824, United States; Plant Resilience Institute, Michigan State University, East Lansing, MI 48824, United States

## Abstract

The increasing frequency and intensity of droughts present significant challenges to global food security. In this study, we examined the genetic and physiological mechanisms underlying drought tolerance and resilience in sorghum (*Sorghum bicolor* L.) by phenotyping the Sorghum Association Panel (SAP; *n* = 397) for a broad suite of traits. These included leaf anatomical characteristics (stomatal density [SD], stomatal size, pore area, stomatal pore area per leaf area, and anatomical maximum stomatal gas conductance), physiological traits [net photosynthetic rate (*A*_n_), stomatal gas conductance (*g*_sw_), and intrinsic water-use efficiency (_i_WUE)], and functional traits (leaf width, leaf thickness, leaf mass area, and chlorophyll content). Substantial natural variation was detected within the SAP, and correlation analyses indicated that leaf anatomical and functional characteristics play key roles in regulating physiological traits, including *A*_n_, *g*_sw_, and *_i_*WUE. Genome-wide association studies identified a genomic hotspot on chromosome 1 (77.5–78.6 Mb) region associated with 3 key single-nucleotide polymorphisms (S01_77550396, S01_78561058, and S01_78619413). Haplotype analysis of these loci uncovered 8 distinct allele combinations influencing SD, *A*_n_, *g*_sw_, and _i_WUE. Application of the Ball–Woodrow–Berry *g*_sw_ model to these haplotypes demonstrated that accessions from haplotypes 1 to 5 exhibited greater stomatal plasticity, displaying more dynamic responses under well-watered conditions. In contrast, accessions from haplotypes 6 to 8 showed more conservative stomatal behavior under water-limited conditions. These results provide insights into the coordinated genetic control of leaf traits underlying drought resilience in sorghum and offer a predictive framework for breeding cultivars with stable performance across diverse water regimes.

## Teaser

Natural genetic variation in leaf anatomical and functional traits drives water-use efficiency and drought resilience in sorghum.

## Introduction

Climate change is increasingly disrupting global agricultural systems, posing a major threat to the productivity of both food and bioenergy crops. Among its various effects, drought or water deficit is one of the most critical constraints to plant growth, directly affecting photosynthesis, water relations, and ultimately reducing crop yield (Seleiman et al. 2021; Ali et al. 2025). Developing climate-resilient crops requires a deeper understanding of the natural genetic variation that governs key morphological, physiological, and anatomical traits associated with water-use and stress adaptation.

Plants cope with drought through both morphological and physiological strategies (Franco-Navarro et al. 2025). Morphological traits such as thicker leaves or denser tissues can reduce water loss (Fang and Xiong 2015; Everingham et al. 2024; Li et al. 2025). Physiological traits, including stomatal gas conductance (*g*_sw_) and net photosynthetic rate (*A*_n_), are central to maintaining carbon gain under stress (Yin and Struik 2009; Asargew et al. 2024). The ratio of *A*_n_ to *g*_sw_, termed intrinsic water-use efficiency (_i_WUE), serves as a key integrative measure of performance under water limitation (Urban et al. 2017; Hatfield and Dold 2019). These physiological processes are strongly influenced by leaf anatomical features such as SD, size, and pore dynamics (Faralli et al. 2019; Fernando et al. 2025).

Stomata, microscopic pores on the leaf surface, regulate gas exchange and water loss. In grasses like sorghum, dumbbell-shaped guard cells flanked by subsidiary cells enable rapid and efficient aperture control (Menezes et al. 2025). Variation in stomatal traits has been documented in major cereals, including wheat, rice, barley, sorghum, and maize (Robertson et al. 2021, 2023; Wall et al. 2022; Caine et al. 2023; Battle et al. 2024; Zhang et al. 2025), and these traits are often tightly coupled with *g*_sw_, *A*_n_, and _i_WUE (Lawson and Blatt 2014; Ferguson et al. 2021; Al-Salman et al. 2024). In C_4_ plants, partial stomatal closure reduces *g*_sw_ and can improve _i_WUE, but at the cost of reduced CO_2_ uptake, creating a trade-off between carbon gain and water conservation (Ozeki et al. 2022; Silva-Alvim et al. 2024).

Modeling *g_sw_* in relation to environmental variables is essential for predicting crop performance under climate change. Empirical models (eg, Jarvis-type) statistically relate *g*_sw_ to environmental factors (Lange et al. 1971; Jarvis 1976; Qi et al. 2023), while semi-empirical models like the Ball–Woodrow–Berry (BWB) model integrate *g*_sw_ as a linear function of *A*_n_, relative humidity (RH) (*H*_s_), and CO_2_ concentration at the leaf surface (*C*_s_) (Ball et al. 1987; Tuzet et al. 2003; Li et al. 2021). A key BWB parameter, the slope (*m* or ball index), reflects the sensitivity of *g*_sw_ to *A*_n_ × *H*_s_/*C*_s_, plus a residual conductance (*g_0_*), which represents *g*_sw_ when *A*_n_ approaches zero. Variation in *m* offers insights into stomatal efficiency and its adjustment under water stress (Ding et al. 2022).

The BWB model has described variations in stomatal behavior through slope (*m*) variability across species under different stresses, including potato and tomato under varying water regimes (Liu et al. 2009; Wei et al. 2018), maize, sunflower, and rice under water stress (Miner and Bauerle 2017; Ding et al. 2022; Qi et al. 2023; Asargew et al. 2024), and rice and sorghum under elevated ozone (Masutomi et al. 2019; Li et al. 2021). Collectively, these studies highlight the adaptability of structure–function relationships governing stomatal patterning and leaf gas exchange.

Sorghum (*Sorghum bicolor* L.), a high-yielding C_4_ grass, is valued for its drought resilience and low input requirements, making it a prime candidate for bioenergy crop improvement (Mullet et al. 2002; Gomez et al. 2025). Despite its adaptive capacity, the genetic architecture underlying variation in leaf-level anatomical and physiological traits linked to drought tolerance, particularly stomatal traits, their integration with physiological performance, and the behavior of BWB model parameters such as the slope (*m*) remains poorly understood. Given the increasing frequency and severity of drought events driven by climate change, the development of accurate, accession-specific models of stomatal conductance (*g*_sw_) is of growing importance.

In this study, we examined natural genetic variation in leaf anatomical traits (eg, SD and size), physiological traits (*A*_n_, *g*_sw_), and functional traits (eg, LW, thickness) in 397 diverse sorghum accessions grown under natural field conditions to identify traits underlying water-use efficiency (_i_WUE). Genome-wide association studies (GWAS) revealed 8 haplotype combinations on chromosome 1 that significantly affected SD, *A*_n_, and *g*_sw_, thereby influencing _i_WUE. One accession from each haplotype was selected for validation of stomatal sensitivity (BWB slope *m*) and _i_WUE under well-watered (WW) and water-stressed (WS) conditions using *g*_sw_-based BWB modeling. Across accessions, *m* consistently declined under WS, but the magnitude of change varied among haplotypes, ultimately affecting _i_WUE. This study represents a comprehensive assessment of natural genetic variation in stomatal traits and their integration with physiological performance across a bioenergy sorghum diversity panel. The findings provide insight into the genetic basis of drought resilience and a foundation for breeding sorghum varieties with improved water-use efficiency.

## Results

### Natural genetic variation in leaf anatomical, physiological, and functional traits in sorghum

**Figure 1:**
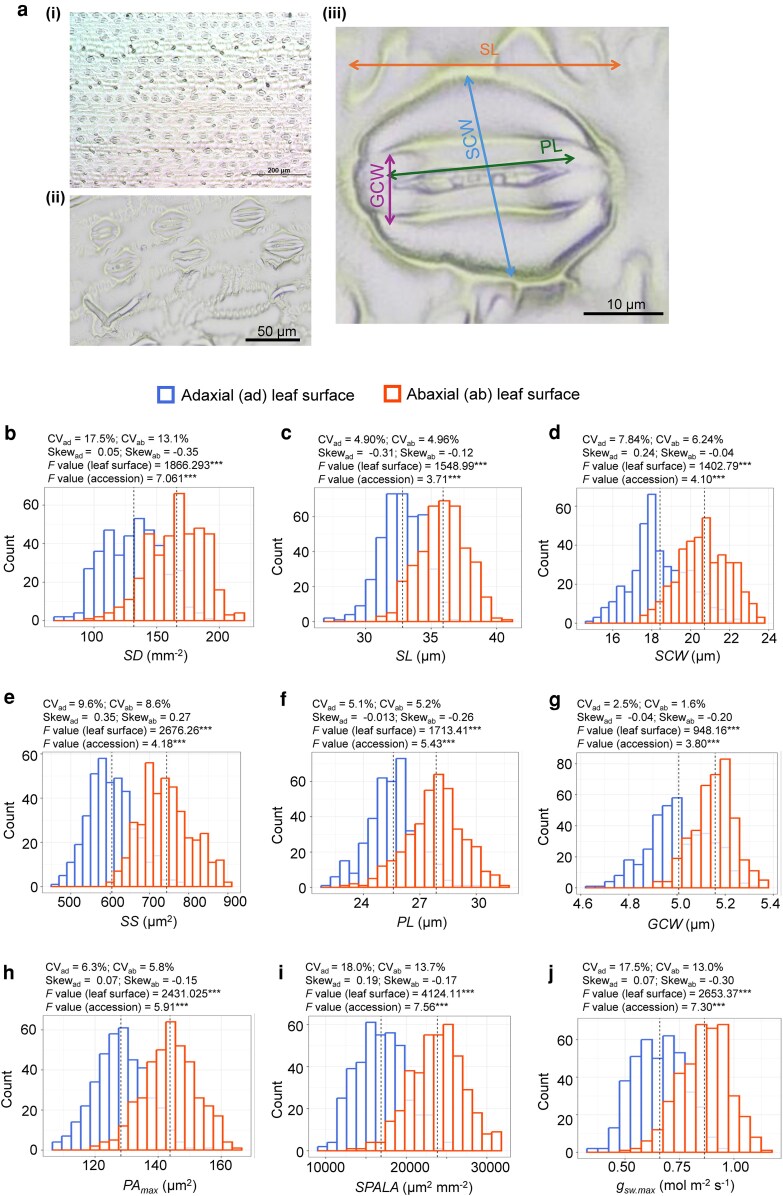
Natural variations in leaf anatomical (stomatal density and size related) traits across sorghum accessions. **(a-i-iii)** Schematic representation of stomata at 10× magnification (scale bar = 200 μm) and 40× magnification (scale bar = 50 μm), along with visualizations of size-related traits (scale bar = 10 μm). **(b–j)** Quantification of stomatal density (SD; mm^−2^), stomata length (SL; μm), stomatal complex width (SCW; μm), stomata size (SS; μm^2^), pore length (PL; μm), guard cell width (GCW; μm), maximum pore area (PA_*max*_; μm^2^), stomatal pore area per leaf area (SPALA; μm^2^ mm^−2^), and anatomical maximum stomatal gas conductance (*g*_sw.max_; mol m^−2^ s^−1^) on the adaxial (ad; upper; blue/left histogram) and abaxial (ab; lower; red/right histogram) leaf surfaces. Dotted lines in each histogram indicate the mean trait value for the respective leaf surface across 397 accessions from the Sorghum Association Panel. For each trait, the coefficient of variation (CV) and skewness (Skew) were calculated separately for each leaf surface. For 2 variable comparisons (eg, between leaf surfaces and among accessions), 2-way ANOVA was performed to obtain *F-* and *P*-values. Asterisks indicate statistical significance: *P* < 0.05*, *P* < 0.01**, *P* < 0.001***.

We conducted comprehensive phenotyping of leaf anatomical, physiological, and functional traits across 397 sorghum accessions from the association panel (Table S1). We first assessed leaf anatomical traits, including SD and a suite of size-related characteristics: stomatal length (SL), stomatal complex width (SCW), stomatal size (SS), pore length (PL), guard cell width (GCW), and maximum pore area (PA_max_). These measurements were performed separately on the abaxial and adaxial surfaces of the leaf (Fig. 1a; Table S2). In addition, we calculated 2 integrative functional metrics such as stomatal pore area per leaf area (SPALA) and anatomical maximum stomatal gas conductance (*g*_sw.max_), as described in previous studies (Wall et al. 2022; Al-Salman et al. 2023a). All anatomical traits exhibited significant variation among accessions and between leaf surfaces, with strong correlations observed between the 2 surfaces, with *R^2^* values ranging from 0.335 to 0.591 (*P* < 0.0001) (Fig. S1). Coefficients of variation (CV) ranged from 1.6% to 18.0%, and the mean values were consistently higher on the abaxial surface compared to the adaxial surface. For example, SD ranged from 100 to 218 mm⁻^2^ on the abaxial side and from 73 to 194 mm⁻^2^ on the adaxial side, reflecting an average increase of 25.5% (Fig. 1b). Similar trends were observed for SS-related traits, with abaxial values exceeding adaxial values by: SL (9.4%), SCW (12.4%), SS (23.1%), PL (8.9%), GCW (3.0%), and PA_max_ (12.1%) (Fig. 1c–h). Likewise, both SPALA and *g*_sw.max_ were significantly greater on the abaxial surface, with average increases of 41.3% and 31.0%, respectively (Fig. 1i and j).

**Figure 2:**
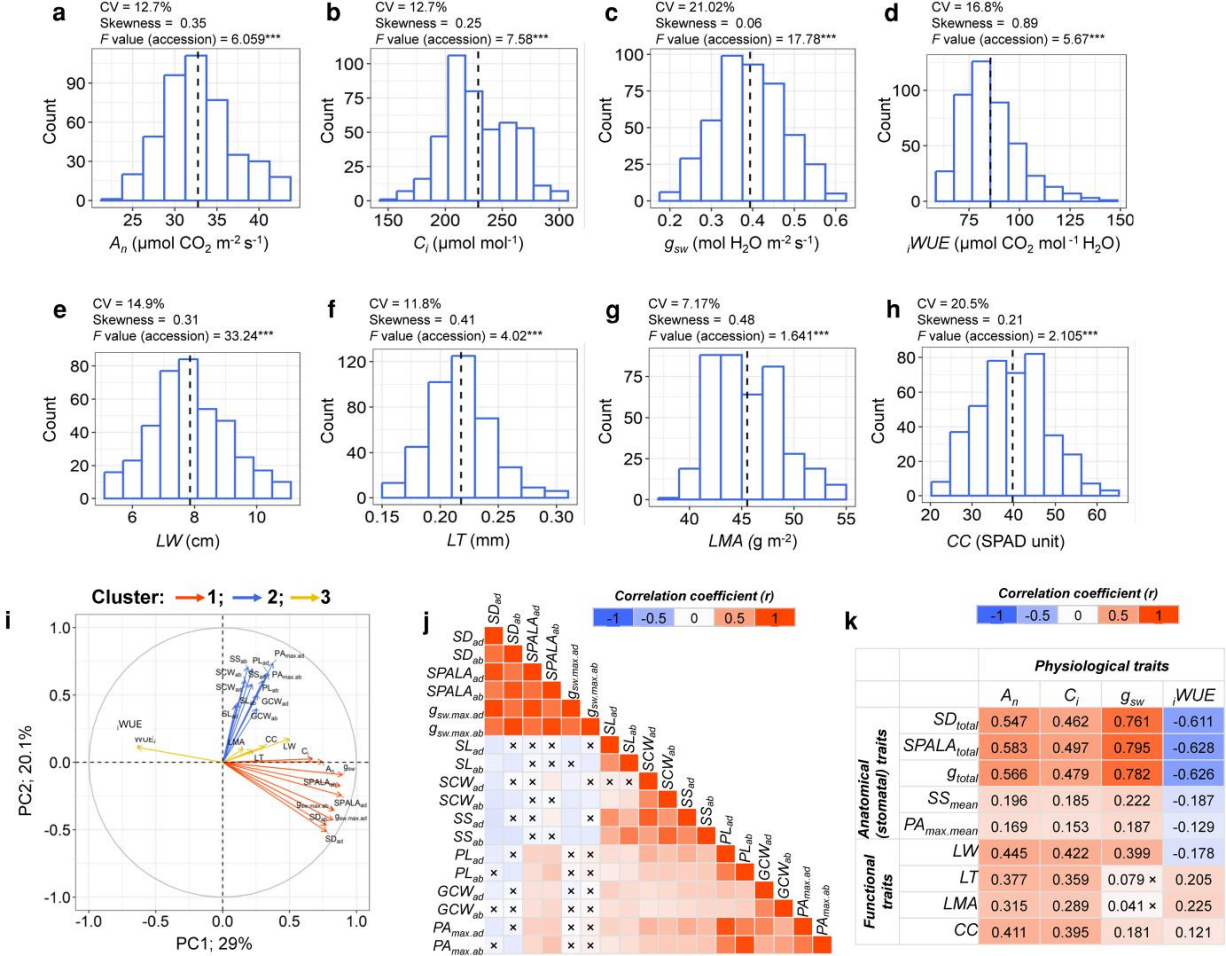
Natural variation in leaf physiological and functional traits across sorghum accessions. **(a–h)** Phenotypic distributions of leaf physiological traits, including net photosynthetic rate (*A_n_*; μmol CO_2_ m^−2^ s^−1^), intercellular CO_2_ concentration (*C*_i_; μmol mol^−1^), stomatal gas conductance to water vapor (*g*_sw_; mol H_2_O m^−2^ s^−1^), and intrinsic water-use efficiency (_i_WUE; μmol CO_2_ mol ^−1^ H_2_O), as well as leaf functional traits, including leaf width (LW; cm), leaf thickness (LT; mm), leaf mass per area (*LMA*; g m⁻^2^), and CC (SPAD units). Dotted line in each histogram indicates the mean trait values across 397 accessions from the Sorghum Association Panel. For each trait, the coefficient of variation (CV) and skewness were calculated. For single-variable comparisons (eg, variation among accessions), 1-way ANOVA was performed to obtain *F-* and *P*-values (*P* < 0.05*, *P* < 0.01**, *P* < 0.001***; ns = not significant). **(i)** PCA with cluster analysis of leaf anatomical, physiological, and functional traits. **(j)** Correlation matrix among stomatal density and size-related traits, based on mean values for each accession on the abaxial (ab) and adaxial (ad) leaf surfaces. **(k)** Correlation matrix between leaf physiological traits with both leaf anatomical (stomatal) and functional traits, based on mean values for each accession. In both correlation analyses, statistically significant correlations (*P* < 0.05) are shown as colored cells, with red indicating positive correlations and blue indicating negative correlations. Color intensity reflects the strength of the correlation coefficient (*r*, ranging from −1 to 1). Nonsignificant correlations are denoted by “×’. See text for abbreviations.

We next evaluated leaf gas exchange parameters, which also displayed significant variation across accessions. CVs ranged from 12.7% to 21.0%, and most traits showed mild positive skewness in their distributions. Specifically, net photosynthetic rate (*A*_n_) ranged from 23.1 to 43.7 μmol CO_2_ m⁻^2^ s⁻^1^, g_sw_ from 0.19 to 0.60 mol m⁻^2^ s⁻^1^, intercellular CO_2_ concentration (*C_i_*) from 147.1 to 305.7 μmol mol⁻^1^, and _i_WUE from 60.3 to 139.96 μmol CO_2_ mol⁻^1^ H_2_O (Fig. 2a–d; Table S2). Lastly, we assessed leaf structural and functional traits, including leaf width (LW), leaf thickness (LT), leaf mass per area (LMA), and chlorophyll content (CC). These traits varied significantly among accessions, with CVs ranging from 7.0% to 20.0%. Trait ranges included LW (5.3–10.8 cm), LT (0.151–0.300 mm), LMA (38.4–54.3 g m⁻^2^), and CC (22.14–64.2 SPAD units) (Fig. 2e–h). Overall, our phenotypic analysis revealed substantial natural genetic variation across the sorghum panel for all traits evaluated, providing a robust foundation for identifying the genetic regulators underlying these traits.

### Leaf anatomical and functional traits determine gas exchange parameters in sorghum

To characterize the structure of variation among leaf anatomical, physiological, and functional traits, we conducted a principal component analysis (PCA). The first 2 principal components (PC1 and PC2) together explained 49.1% of the total variance, with PC1 accounting for 29% and PC2 contributing 20.1% (Fig. 2i). Complementing the PCA, a cluster analysis based on the PCA grouped traits into 3 distinct clusters. Cluster 1 was primarily composed of traits related to SD and physiological parameters (eg, *A*_n_, *g*_sw_, *C*_i_), along with anatomical gas conductance. Cluster 2 was predominantly associated with SS features, such as PL and GCW, while cluster 3 included leaf functional traits and _i_WUE (Fig. 2i).

We then performed a comprehensive correlation analysis to detail the relationships among the phenotyped traits (Fig. 2j). Within anatomical (stomatal) traits, SD was weakly but significantly negatively correlated with SS-related traits on corresponding surfaces. Conversely, all SS traits (SL, SCW, SS) exhibited strong positive correlations with each other on both leaf surfaces. SPALA exhibited positive correlations with both SD and pore-related features (PL, GCW, and PA_max_); however, the magnitude of correlation was greater with SD than pore-related features, suggesting the former has a greater influence on SPALA than the latter (Lawson et al. 1998). Similarly, anatomical g_sw.max_, which integrates SD and pore size-related traits, was most positively associated with SD on both leaf surfaces. No significant correlations were observed between *g*_sw.max_ and either PA_max_ or GCW, reinforcing the primary influence of SD on anatomical *g*_sw.max_, consistent with previous findings (Wall et al. 2022).

Among leaf physiological traits, *A*_n_, *g*_sw,_ and *C*_i_, were strongly intercorrelated, reflecting coordinated regulation of photosynthesis and gas exchange. In contrast, _i_WUE was strongly negatively correlated with *g*_sw_ (*r* = *−0.802; P*  *<* 0.05), highlighting a trade-off between water conservation and gas exchange capacity (Fig. S2). All leaf functional traits exhibited positive correlations (Fig. S3), with LT showing particularly strong associations with LMA and CC, suggesting shared developmental or structural determinants.

To elucidate the integrative effects of leaf anatomical (stomatal) and functional traits on physiological performance, correlation analyses were conducted on combined trait metrics (Fig. 2k). For this analysis, stomatal features (SD, SPALA, *g*_sw.max_) were summed across both leaf surfaces to obtain SD_total_, SPALA_total,_ and *g*_total_. Mean SS (*SS*_mean_) and mean pore area (PA_max.mean_) were also calculated across both leaf surfaces. Significant positive correlations were observed between stomatal traits and gas exchange parameters, particularly *A*_n_, *C_i_*, and *g*_sw_. Among the stomatal traits, the strongest correlations were detected for SPALA_total_, followed by *g*_total_, and SD_total_ and lowest for SS_mean_ and PA_max.mean_. In contrast, all stomatal traits were negatively correlated with *_i_*WUE, indicating a trade-off between gas exchange capacity and water conservation. Regarding leaf functional traits, thicker leaves, greater LMA, and higher CC exhibited significant positive correlations with *A*_n_, *C*_i_, *g*_sw_, and *_i_*WUE, whereas LW was negatively associated with _i_WUE (Fig. 2k). Overall, the results highlight key anatomical and functional traits that underpin physiological performance and influence water-use efficiency in sorghum.

### Genome-wide association mapping identified resilience allele haplotypes for improved iWUE

**Figure 3:**
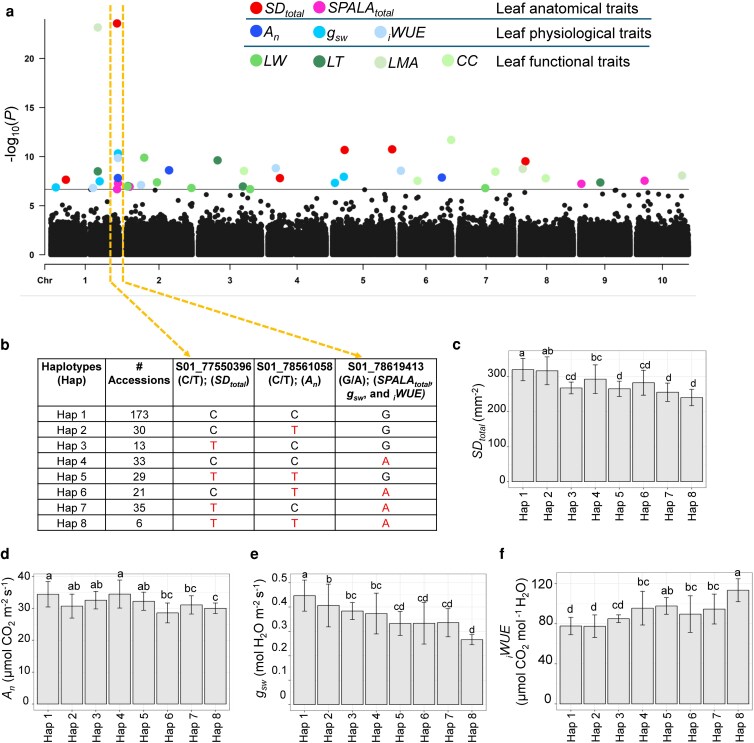
Genome-wide association studies (GWAS) on leaf anatomical, physiological, and functional traits in sorghum. **(a)** GWAS of leaf anatomical (SD_total_ and SPALA_total_), physiological (*A*_n_, *g*_sw_, and iWUE), and functional traits (LT, LW, LMA, and CC) across accessions from the Sorghum Association Panel. Significant SNPs were identified using the FarmCPU-based GWAS method. The plot shows -log10(*P*-values) of SNPs on the y-axis, with SNPs ordered by chromosomal location on the *x*-axis. A gray horizontal line indicates the significance threshold (2.13 × 10^−7^). **(b)** Haplotype allele analysis of significant SNPs presents on chromosome 1 (77.5–78.6 Mb) associated with multiple traits. Reference and alternative alleles are shown in black and red, respectively. **(c–f)** Quantification of SD_total_, *A*_n_, *g*_sw_ and _i_WUE among different haplotype allele combinations. Data represent mean trait values ± SD for accessions carrying each allele combination. Lowercase letters above each bar indicate significant differences among the haplotypes, determined by 1-way ANOVA with Tukey's HSD post hoc test (*P*  *<* 0.05*)*. See text for abbreviations.

We performed GWASs to identify genetic regulators of leaf anatomical (SD_total_ and SPALA_total_), physiological (*A*_n_, *g*_sw_, and _i_WUE), and functional traits (LW, LT, LMA, and CC) in sorghum (Fig. 3a and Fig. S4). A total of 40 single-nucleotide polymorphisms (SNPs) were significantly associated with these traits (Table S3). These SNPs were closely located near 401 genes (75 kb upstream and 75 kb downstream), several of which encode components of photosynthetic pathways, transporters, hormone signaling and biosynthesis, and transcription factors (Table S4). Notably, 13 out of these 401 genes harbored SNPs within their genomic regions (Table S4).

Among the 40 significant SNPs, 10 were associated with anatomical traits, 11 with physiological traits, and 17 with functional traits (Table S4). Two SNPs, S01_55126705 and S01_78619413, both carrying G/A alleles, exhibited pleiotropic effects across multiple traits. Locus S01_55126705 was associated with both *LT* and *LMA*, and sorghum accessions carrying the alternative allele, showing higher values for these traits compared to those with the reference allele (Fig. S5). In contrast, locus S01_78619413 was associated with SPALA_total_, *g*_sw_, and _i_WUE, where accession harboring the alternative alleles exhibited lower SPALA_total_ and *g*_sw_ but increased _i_WUE (Fig. S6).

To further explore potential resilience-associated alleles, we identified a genomic hotspot on chromosome 1, which harbored 9 out of the 40 significant SNPs (∼23%). Correlation analysis among these loci revealed a positive association, particularly within the 77.5–78.6 Mb region encompassing S01_77550396 (C/T; associated with SD_total_), S01_78561058 (C/T; associated with *A*_n_), and S01_78619413 (pleiotropic locus; G/A; SPALA_total_, *g*_sw_, *_i_*WUE) (Fig. 3b; Fig. S7). At individual allele level, the alternative allele of these SNPs displayed reduced performance for SD_total_, *A*_n_, SPALA_total_, *g*_sw_ but with improved _i_WUE (Figs. S6, S8, and S9). When analyzed in combination, these 3 loci defined 8 distinct haplotypes, each comprising a specific allelic configuration and represented by varying numbers of sorghum accessions. Hap 1 (CCG), composed of reference alleles of all 3 SNPS, was the most frequently observed, found in 173 accessions within the association panel. This haplotype resulted in a greater impact on SD_total_, *A*_n_, and *g*_sw_, albeit with a reduced impact on _i_WUE (Fig. 3c–f). Hap 2 (CTG), Hap 3 (TCG), and Hap 4 (CCA), each containing one alternative allele, showed a moderate decrease in SD_total_, *A*_n_, and *g*_sw_, leading to slight improvements in _i_WUE (Fig. 3c–f). Hap 5 (TTG), Hap 6 (CTA), Hap 7 (TCA), with 2 alternative alleles, exhibited a significant reduction in SD_total_, *A*_n_, and *g*_sw_, which resulted in significant improvements in *_i_*WUE (Fig. 3c–f). Finally, Hap 8 (TTA), consisting of alternative alleles for all 3 SNPs, displayed the highest _i_WUE, although this was accompanied by a reduction in SD_total_, *A*_n_, and *g*_sw_ (Fig. 3c–f). Collectively, these results identify distinct haplotypes with potential utility for improving water-use efficiency in sorghum breeding while maintaining acceptable growth performance.

### Accessions carrying resilience allele haplotype combinations demonstrated a higher capacity to maintain *g*_sw_ under water stress conditions

We then evaluated the performance of these haplotypes using the BWB model, in which stomatal conductance (*g*_sw_) is described as a function of net photosynthetic rate (*A*_n_), RH at the leaf surface (*H*_s_), and CO_2_ concentration at the leaf surface (*C*_s_), collectively referred to as the ball index (*A*_n_  *×*  *H*_s_*/C*_s_). Eight accessions, each representing a distinct haplotype combination, were selected, and the BWB model was applied under WW and WS conditions in controlled growth chamber experiments. The effectiveness of the WS treatment was confirmed by a reduction in the quantum yield of primary photochemistry (*F*_v*/*_*F*_m_) in dark-adapted leaves (Fig. S10). For BWB modeling, *A*_n_ and *g*_sw_ were recorded across a range of light intensities, with consistently higher values observed under WW conditions compared to WS across all accessions (Fig. S11).

**Figure 4:**
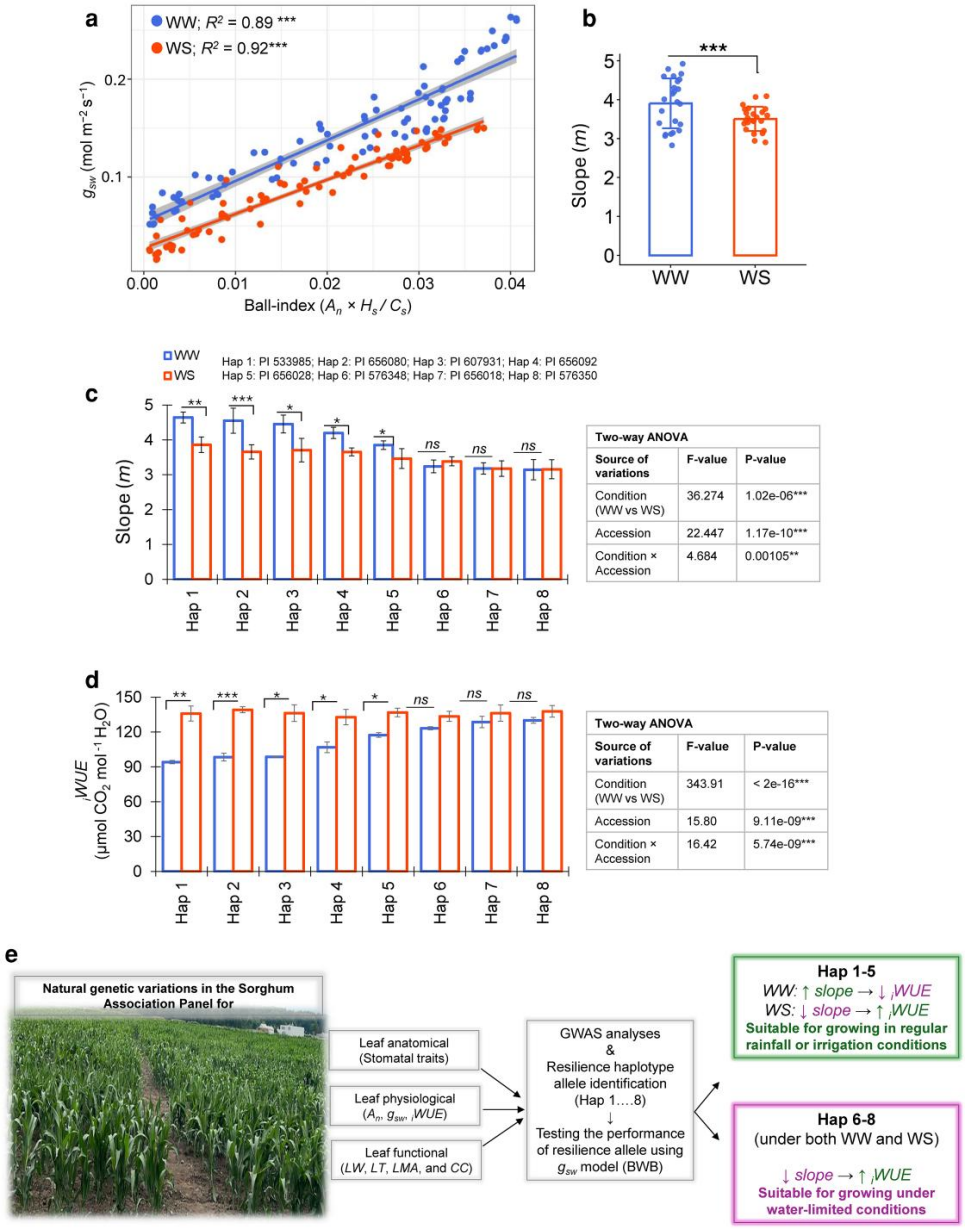
Stomatal gas conductance modeling of selected sorghum accessions across haplotype allelic combinations under well-watered (WW) and water-stressed (WS) conditions. **(a)** Regression analysis between *g*_sw_ and ball index (*A*_n_  *×*  *H*_s_*/C*_s_) from the Ball–Woodrow–Berry (BWB) model under WW and WS conditions. Each point represents an individual measurement at a given PPFD level. A total of 88 points are shown for WW and 88 points for WS, representing 11 PPFD levels measured across 8 accessions, with values averaged from 3 biological replicates per accession. Solid lines represent linear regression fits for each treatment (blue, WW; red, WS), with associated *R^2^* values. Shaded areas indicate the 95% confidence intervals around the mean fitted lines and are shown for visualization purposes. **(b)** Overall estimation of the slope (*m*) parameter under WW and WS conditions. The bar plot with jitter shows individual slopes for each accession, with 24 data points per treatment (8 accessions × 3 biological replicates per accession). Statistical significance between WW and WS was assessed using Student's *t* test (*P < 0.05, **P < 0.01, ***P < 0.001). **(c, d)** Accession-wise visualization of slope (*m*) and intrinsic water-use efficiency (_i_WUE) across 8 representative sorghum accessions under WW and WS conditions. Values represent mean ± standard deviation (SD) from 3 biological replicates per accession. Statistical significance between WW and WS for each accession was assessed using Student's *t*-test (*P < 0.05, **P < 0.01, ***P < 0.001). Overall effects of accession, treatment, and their interaction were evaluated using 2-way ANOVA. **(e)** Graphical summary of the study. Natural genetic variation in leaf anatomical, physiological, and functional traits identified haplotype-specific allele combinations through GWAS. Graphical summary of the study. Natural genetic variation in leaf anatomical, physiological, and functional traits identified haplotype-specific allele combinations through GWAS. A Ball–Woodrow–Berry (BWB) stomatal conductance model assessed the performance of representative accessions from each haplotype combination, highlighting their suitability for cultivation under either regular rainfall/irrigated or water-limited conditions.

We next evaluated the relationship between the ball index and *g*_sw_, which revealed a positive correlation under both WW and WS conditions (Fig. 4a). However, the magnitude of both the ball index and *g*_sw_ was significantly higher under WW compared to WS conditions. To assess stomatal responsiveness, we examined the overall mean values of the slope parameter (*m*) across selected accessions from the BWB model. The *m* values were significantly higher under WW than WS conditions (Fig. 4b). When analyzed individually, accessions corresponding to haplotypes 1 through 5 showed higher *m* values under WW conditions, while haplotypes 6 through 8 exhibited lower *m* values. Under WS conditions, *m* significantly decreased in haplotypes 1 to 5 but remained unchanged in haplotypes 6 to 8 (Fig. 4c).

We further explored the overall relationship between *m* and iWUE, which displayed a strong negative correlation (*r* = *−*0.721, *P* < 0.001), indicating that higher stomatal activity/sensitivity (greater *m*) was associated with lower iWUE (Fig. S12). At the accession level, those from haplotypes 1 to 5 exhibited significantly lower *i*WUE under WW conditions, similar to patterns observed in the field (Fig. 4d). However, under WS conditions, these accessions showed an increase in iWUE, likely due to a sharp decline in slope (*m*). In contrast, accessions from haplotypes 6 to 8 maintained moderately higher _i_WUE under both WW and WS conditions (Fig. 4d).

Overall, our findings support the presence of a stable, conservative water-use strategy that is consistently observed across both fields and controlled environments. Specifically, accessions from haplotypes 1 to 5 exhibit a higher degree of stomatal plasticity, characterized by more dynamic responses to changes in water availability. In contrast, accessions from haplotypes 6 to 8 demonstrate a more conservative and less plastic stomatal behavior, maintaining relatively stable slope (*m*) values regardless of environmental conditions.

## Discussion

Sorghum is a vital crop for global food security, serving as a staple food for over half a billion people worldwide and providing a gluten-free source of carbohydrates, protein, and fiber. Beyond its role as human food, sorghum is an emerging bioenergy crop, offering high biomass yields and serving as a renewable feedstock for bioethanol production (Stamenković et al. 2020). While sorghum is considered a drought-tolerant crop, it can still experience significant yield losses under water-limited conditions. However, the increasing frequency and intensity of drought events due to climate change pose a significant threat to sustainable sorghum production (Heino et al. 2023; Gardi et al. 2025).

Plants, being sessile organisms, have evolved a suite of mechanisms to mitigate the effects of drought stress, including avoidance, recovery, survival, and tolerance strategies (Nour et al. 2024). These mechanisms involve complex biological processes, encompassing physiological, biochemical, genomic, proteomic, and metabolomic changes (Seleiman et al. 2021). For instance, plants can survive drought stress by exhibiting phenotypic plasticity in their root systems, regulating stomatal closure, and displaying drought-adaptive traits such as leaf rolling, stem waxiness, and the maintenance of green leaf area (stay green) (Alongi et al. 2025; Saini et al. 2025). Furthermore, efficient water use is crucial for drought tolerance (Qiao et al. 2024). Therefore, understanding the genetic variation in leaf anatomical, physiological, and functional traits under natural environmental conditions is critical for developing sustainable crop production strategies in the context of climate change, in which drought-responsive traits may have gradually adapted and evolved over time.

### Stomatal density and size features contribute to gas exchange and photosynthesis

Stomata, the microscopic pores on the leaf surfaces, serve as dynamic gatekeepers for carbon assimilation and water loss, linking the carbon and hydrological cycles at the leaf level (Qi and Torii 2018; Haworth et al. 2021). Through their regulation of stomatal conductance to water vapor (*g*_sw_), they mediate the influx of CO_2_ required for photosynthesis (*A*_n_) while controlling transpirational water loss (Lawson and Blatt 2014). In sorghum, leaves are amphistomatous, bearing stomata on both adaxial and abaxial surfaces, with higher density typically observed abaxially, a pattern confirmed in our results and consistent with previous studies (Bheemanahalli et al. 2021).

Our results demonstrated a positive relationship between total SD and *g*_sw_, suggesting that accessions with more numerous stomata can potentially achieve higher CO_2_ uptake rates and faster growth under optimal conditions (Fig. 2k). Interestingly, we observed a weak but negative correlation between SD and size-related traits on both surfaces, consistent with physical and developmental constraints where increased size often compensates for reduced density (Lawson and Morison 2004). Although both density and size influenced *A*_n_ and *g*_sw_, density exerted a greater effect, suggesting that manipulation of SD could be a more effective lever for altering gas exchange in sorghum. However, such manipulations are likely constrained by compensatory adjustments in SS and operational behavior, as previously noted in *Arabidopsis* (Doheny-Adams et al. 2012; Dittberner et al. 2018).

Our study further demonstrated that SD and pore-related features significantly influence total leaf anatomical *g*_sw.max_ (*g*_total_) obtained from both surfaces, which translates into accession-specific operational or physiological *g*_sw_ (McElwain et al. 2016). Although *g*_sw_ and *g*_total_ are not same, the strong empirical relationship between them (*r*  *= 0.782; P*  *<* 0.05), suggests that *g*_total_ can be used as a proxy for predicting *g*_sw_ in sorghum accessions varying in stomatal anatomical traits (Fig. 2k) (Muir 2020; Al-Salman et al. 2023a). Discrepancies between these measures may flag impaired stomatal function, making this approach a potential screening tool for identifying mutants or stress-affected plants with altered stomatal responsiveness (McElwain et al. 2016).

### Stomatal density and size features affect intrinsic water-use efficiency

_i_WUE, defined as the ratio of *A*_n_ to *g*_sw_, is a key parameter for quantifying the carbon gain per unit of water lost at the leaf surfaces (Leakey et al. 2019). In our dataset, *g*_sw_ was negatively correlated with _i_WUE, such that accessions with low *g*_sw_ (and thus lower *A*_n_) exhibited higher _i_WUE, and vice versa (Fig. 2k). This aligns with the well-documented physiological trade-off between maximizing photosynthetic carbon gain and conserving water (Stinchcombe et al. 2010). Since *g*_sw_ is regulated through stomatal traits, they play an important role in the acclimation of plants. A negative relationship between SD and _i_WUE emerged from our analysis, consistent with previous reports in diverse species, including rice (Caine et al. 2019), wheat (Dunn et al. 2019), barley (Hughes et al. 2017), maize (Zhao et al. 2015), and sorghum (Al-Salman et al. 2023a and b), where reduced density is associated with increased water-use efficiency and, in many cases, enhanced drought tolerance.

SS is also linked to functional dynamics. Smaller stomata tend to open and close more rapidly in response to environmental fluctuations, allowing tighter control of *g*_sw_ and minimizing transpirational water loss during transient stress events (Drake et al. 2013; Lawson and Vialet-Chabrand 2019). Larger stomata, by contrast, may support higher maximum conductance but often at the cost of slower response times, potentially leading to inefficient water-use under variable conditions (Lawson and Blatt 2014; Al-Salman et al. 2023b). In our study, a negative relationship between mean SS and _i_WUE supports the hypothesis that smaller stomata enhance water conservation through faster closure kinetics (Fig. 2k). From an evolutionary perspective, SS and density are thought to be shaped by atmospheric CO_2_ history, with low-CO_2_ environments selecting for faster stomatal closure to maintain internal CO_2_ while reducing water loss (Elliott-Kingston et al. 2016).

### Leaf functional traits affect photosynthesis and water-use efficiency

Leaf functional traits such as LW, LT, LMA, and CC play vital roles in regulating photosynthesis, water-use efficiency, and crop yield (Afzal et al. 2017; Puangbut et al. 2017; Cano et al. 2019; Pan et al. 2022). For instance, LW is associated with drought tolerance and water-use efficiency in C_4_ plants (Pan et al. 2022; Struik and Driever 2022). Narrower leaves generally exhibit lower photosynthetic rates but higher water-use efficiency, consistent with our observations and previous reports in sorghum and maize (Cano et al. 2019; Zhi et al. 2022). In contrast, wider leaves, which often have lower vein density, require higher stomatal conductance to sustain photosynthesis, leading to increased water loss and reduced water-use efficiency (Evans et al. 2009; Pan et al. 2022). LT is another important anatomical trait influenced by leaf cell structure and arrangement. It serves as an indicator of drought stress due to its sensitivity to water availability and vapor loss. In C_4_ plants, LT reflects the coordinated arrangement of bundle sheath and mesophyll cells without differentiation into palisade and spongy tissues (Retta et al. 2023). In our study, thicker leaves were positively correlated with both *A*_n_ and _i_WUE (Fig. 2k). These relationships may be attributed to increased chloroplast density, greater internal leaf volume, a thicker boundary layer (Palliotti et al. 1994), and a protective cuticular wax layer (Xue et al. 2017), all of which contribute to enhanced photosynthesis and reduced water loss.

LMA, a composite trait determined by leaf thickness and tissue density, integrates anatomical (epidermis, mesophyll, vascular, and sclerenchymatic tissues) and chemical (carbon-rich vascular and nitrogen-rich mesophyll tissues) characteristics (Poorter et al. 2009). Our findings revealed a positive correlation between LMA and both *A*_n_ and _i_WUE, suggesting that higher LMA, likely resulting from increased mesophyll content, may promote CO_2_ uptake and photosynthesis. Additionally, the presence of lignified tissues associated with elevated LMA may enhance leaf toughness and improve stress resilience (Alvarez-Clare and Kitajima 2007). CC also showed positive associations with *A_n_*, g_sw_, and _i_WUE. This may reflect its role in driving photosynthesis while differentially influencing stomatal behavior (Ghannoum 2008).

Taken together, these results highlight the integrative role of leaf functional traits in modulating photosynthetic performance and water-use efficiency. Further research combining leaf-level measurements with whole-plant water and carbon flux analyses, as well as genetic dissection of trait variability, will be essential for leveraging these traits in sorghum improvement programs.

### Accessions carrying resilience allele haplotypes tolerate water stress conditions

Our genome-wide association analysis identified several previously unidentified and previously reported regulators of sorghum leaf anatomical (stomatal), physiological, and functional traits (Table S4). Among the previously unidentified regulators, SNPs associated with stomatal traits (S01_17830866, S01_77550396, S08_6468928, S01_77672597, and S09_2308492) were located within the genomic regions of Sobic.001G197500, Sobic.001G507800 (Subtilisin), Sobic.008G060500 (leucine-rich repeat protein), Sobic.001G508800 (β-amylase), and Sobic.009G025950, respectively. Functional validation of these candidate genes is required to determine their specific roles in stomatal regulation.

Among the known regulators, SNPs associated with *A*_n_ (S01_78561058, S02_50622890, S06_49207918) were located near genes crucial for photosynthesis, including NADH dehydrogenase (Sobic.001G519000), glyceraldehyde-3-phosphate dehydrogenase (Sobic.001G519800), glucose-6-phosphate dehydrogenase (Sobic.006G126300), and malate synthase (Sobic.006G127100). These genes are linked to sugar and amino acid metabolism (Muñoz-Bertomeu et al. 2009), the Calvin–Benson cycle (Preiser et al. 2020), cyclic electron transport in photosynthesis (Ma et al. 2021), and glyoxylate cycle, which enables the conversion of steroid lipid into carbohydrate (Pua et al. 2003). We also identified an aquaporin gene (Sobic.005G091600; SIP1-1), colocalized with a SNP associated with *g*_sw_ (S05_13529041). Aquaporins are a class of channel-forming proteins that facilitate the diffusion of various small solutes, including water and CO_2,_ into guard cells (Kadam et al. 2017). In addition, SNPs associated with iWUE (S04_9532537) were closely linked to Auxin response factor (Sobic.004G102301) and BES1/BZR1 (Sobic.004G102500) plant transcription factors, both of which are known in *Arabidopsis* to regulate root system architecture, thereby enhancing water uptake and promoting growth under stress conditions (Marin et al. 2010; Al-Mamun et al. 2024).

Our study further identified a hotspot region on chromosome 1 (77.5–78.6 Mb) containing 3 highly correlated SNPs (S01_77550396, S01_78561058, S01_78619413), colocalized with genes encoding cell division proteins (Sobic.001G508000, Sobic.001G508300), NAD-dependent epimerase (Sobic.001G519400), 1,3-beta-glucan synthase (Sobic.001G521500), and many more genes (Fig. 3b; Table S4). Haplotype analysis revealed resilient allele combinations within this region that substantially influence total SD, photosynthetic capacity, and overall water-use efficiency under natural field conditions (Fig. 3c–f). These combinations likely represent coordinated genetic architectures conferring adaptive advantages in variable environments, although the functional roles of the underlying alleles remain to be experimentally validated.

### Linking genetic haplotypes to physiological performance under water stress

To complement genetic associations with functional outcomes, we evaluated the physiological performance with a focus on water-use efficiency of 8 accessions from each haplotype group under different water regimes (WW and WS conditions) in a controlled growth chamber. Specifically, we assess the empirical slope (*m*) representing the relationship between *g_sw_* to BWB model's ball index, accounting *A*_n_, *H*_s_, and *C*_s_ on the leaf surface. Although alternative models such as the Medlyn model (MED) are available, previous studies in maize (Yun et al. 2020) and in sorghum under elevated ozone (Li et al. 2021) have shown minimal performance differences between BWB and MED models. The BWB model can utilize data from light, CO_2_, temperature, and humidity response curves (Collatz et al. 1991); however, we focused solely on the light response curve because the stomatal response to changes in photosynthetic photon flux density (PPFD) in C_4_ plants is generally smaller compared to C_3_ plants (Miner and Bauerle 2017). This is likely due to the morphology and mechanics of the dumbbell-shaped stomata of C_4_ grasses, which enhance their ability to track environmental changes more effectively than non-grass species (Hetherington and Woodward 2003; Franks and Farquhar 2007; Nunes et al. 2022).

Exposure to water-stress resulted in decreased *A*_n_ and *g*_sw_ across different PPFD ranges in sorghum accession from different haplotype groups, leading to a significant reduction in the ball index (Fig. 4a). Previous studies have shown inconsistent effects of stress on *m* parameter of the BWB model, with some studies reporting no change under elevated ozone (Li et al. 2021), water stress (Miner and Bauerle 2017) or elevated CO_2_ (Leakey et al. 2006; Ainsworth and Rogers 2007) in sorghum, sunflower, and soybean, respectively. In contrast, some studies reported a significant decrease in *m* under drought conditions in rice (Asargew et al. 2024) and elevated CO_2_ conditions in soybean and wheat (Bunce 2004; Tausz-Posch et al. 2012; Tausz et al. 2013).

In our study, accessions from haplotype groups 1 to 5 exhibited higher *m* values under WW conditions compared to those from groups 6 to 8 (Fig. 4c). The higher *m* values in these accessions were likely due to their higher SD, which facilitates optimal gas exchange and less water-use efficiency under non-stressful conditions. In the context of breeding, accessions from haplotype groups 1–5 may be well-suited for regions with regular rainfall or irrigation (Miner and Bauerle 2017). Furthermore, the higher *m* values under WW conditions indicate a greater sensitivity of stomata to environmental factors such as radiation, humidity, soil water availability, and CO_2_ concentration. Interestingly, under water stress, accessions from haplotype groups 1 to 5 may experience partial or complete stomatal closure, restrict gas exchange, and lead to a reduced slope parameter. The reduction in *A*_n_ caused by water stress may also result from non-stomatal limitations, such as a decrease in the maximum carboxylation rate and maximum electron transport rate, which would reduce carbon assimilation (Zargar et al. 2017). In contrast, accessions from haplotype groups 6 to 8 exhibited no significant changes in *m* values under either WW or WS conditions. This stability in the slope (*m*) indicates a restrained gas exchange strategy and reflects a more conservative (efficient water-use), less plastic stomatal behavior, which may enhance drought tolerance (Fig. 4d and e). Therefore, these accessions may be better suited for cultivation in water-limited environments.

## Conclusion

This study reveals substantial natural genetic variation in stomatal traits, leaf physiological characteristics, and functional traits within the sorghum population. SD, SS-related traits, and leaf functional traits play crucial roles not only in regulating stomatal conductance (*g*_sw_) but also in influencing net photosynthetic rate (*A*_n_) and intrinsic water-use efficiency (*_i_*WUE). GWASs identified key genomic regions associated with these traits, particularly a hotspot on chromosome 1. Haplotype analysis within this region uncovered distinct allele combinations across 8 haplotypes that significantly affect SD, *g*_sw_, *A*_n_, and _i_WUE. Furthermore, stomatal conductance modeling suggests that accessions carrying specific haplotypes may be better adapted for cultivation under either WW or water-limited conditions (Fig. 4e). Collectively, these findings advance our understanding of the genetic architecture underlying drought resilience in sorghum and provide a foundation for breeding climate-resilient cultivars capable of thriving across diverse water availability scenarios.

## Materials and methods

### Site description, plant material, and growth conditions

A total of 397 accessions from the Sorghum Association Panel (SAP) were utilized to capture the global natural variation in leaf anatomical, physiological, and functional traits (Table S1) (Casa et al. 2008; Ortiz et al. 2017; Singh et al. 2025). The field experiment was conducted during the summer growing season (June to October 2022) at the Michigan State University research field, located at 42.7370° N latitude, 84.4839° W longitude, and an elevation of approximately 261 m above sea level. The soil in the research field was classified as loamy with high available water-holding capacity and moderate or moderately slow permeability. According to Koppen–Geiger Climate Classification, the experimental field has a “Dfb” subtype of climate (warm summer, snowy winter, and fully humid) with an average annual precipitation of ∼760 mm with most of the precipitation received between April and June (∼85 mm).

Prior to planting, the soil was treated with 23.6 ml/meter^2^ of starter fertilizer containing 16.55% N, 6.59% P_2_O_5_, and 2.03% K_2_O. The entire SAP panel with nearly 10% replication of BTx623 (PI 564163) as checks was grown in a 1.15-acre experimental field. Plants were grown using a randomized complete block design, with blocks organized into rows and columns. Each accession was planted in 2 rows (3.05 meters long) with rows spaced 76.2 cm, with a 91 cm alley. An Almaco precision planter was used to plant 41 seeds per row (82 seeds per accession) 7.6 cm apart and ∼ 4.0 cm deep in the soil on 6 June 2022. Ten to twelve well-grown plants from the center of each row were selected for the quantification of multiple traits, while the outer plants in each row were treated as border plants and were excluded from data collection. After the seeds were planted in the experimental field, daily weather data were monitored using the Weather Underground station located within 1 km of the experimental site. https://www.wunderground.com/weather/us/mi/meridian-charter-township/KMIMERID11.

### Leaf anatomical (stomatal features), physiological (gas exchange), and functional traits measurements

Leaf anatomical (stomatal features), physiological (gas exchange), and functional traits were measured in the SAP during the booting stage, defined as the developmental phase when the panicle remains enclosed within the flag leaf sheath, and all other leaves are fully expanded. Although the flag leaf is the final leaf during the boot stage, it is often shorter than the preceding leaf. Therefore, to ensure consistency and adequate leaf surface area across accessions, the middle portion of the topmost fully expanded collar leaf was selected for trait measurements. Due to variation in developmental timing among accessions, not all plants reached the booting stage simultaneously. Therefore, phenotyping was conducted in 4 temporal batches, based on days after sowing: super-early booting (< 60 days), early booting (61–66 days), intermediate booting (66–72 days), and late booting (73–85 days). Average daily temperature (AveT; °C) and average humidity (AveH; %) on each sampling day are provided in Fig. S13.

#### Leaf anatomical (stomata) trait measurements

Various stomatal traits such as SD, SS, and maximum pore area (PAmax) were quantified using the nail paint impression method (Fig. S14a), following the protocol described by Al-Salman et al. (2023a). Impressions were collected in multiple batches, aligned with the phenotyping schedule outlined above, on clear sunny days between 9.00 and 10.00 AM to minimize environmental variation. For each accession, 3 plants were sampled, and impressions were taken from both the adaxial (upper) and abaxial (lower) leaf surfaces. Leaves were gently cleaned with lint-free tissue paper, then a thin layer of clear nail polish (Insta-Dri Top Coat, Sally Hansen, USA) was applied over an area approximately 10 × 15 mm. After air drying for 5 min, the film was carefully peeled from the leaf surface and stored in labeled containers for subsequent analysis.

Once all samples were prepared, each peel was mounted on a glass slide, covered with a coverslip, and gently pressed using fine-point tweezers. Stomatal observations were performed using an OLYMPUS IX71 Differential Interference Contrast (DIC) microscope equipped with a DP Controller image capture system. For measuring SD, images were captured under 10× magnification, and 2 fields of view per leaf impression were counted using ImageJ software (bundled with Java 1.8.0_112, National Institutes of Health, USA). The number of stomata was then divided by 0.59 mm^2^ (area of each field of view) to estimate SD (mm^−2^). SD was calculated separately for the adaxial leaf surface (SD_ad_) and abaxial leaf surface (SD_ab_), and the total SD_total_ was calculated using SD_total_ = SD_ad_ + SD_ab_.

For quantification of SS and PA_max_, impressions from both surface of leaves (3 impressions per accessions) were imaged under 40× magnification. Then, 4 to 5 stomata per impression were randomly selected and scored for SL (μm), SCM (including 2 guard cells and 2 subsidiary cells; μm), PL (μm), and pore width or GCW (μm) using ImageJ software. As the shape of a fully open stomatal pore geometrically fits a rectangular shape in grasses (Franks and Farquhar 2007; Franks et al. 2014), SS and PAmax were calculated as the equation given below, expressed in μm^2^.


SS=SL×SCW

$$PA_{\max}=\mathrm{PL}\times \mathrm{GCW}$$

We also determine stomatal pore area per leaf area (SPALA; μm^2^ mm^−2^) as the equation given below:


$$\mathrm{SPALA}=PA_{\max}\times \mathrm{SD}$$

SPALA from adaxial leaf surface (SPALA_ad_) and abaxial leaf surface (SPALA_ab_) were calculated separately. Then, total SPALA (SPALA_total_) for each accession was calculated as the sum of SPALA_ad_ and SPALA_ab_.

Leaf anatomical maximum stomatal gas conductance to water vapor (*g*_sw.max_; mol m^−2^ s^−1^) was calculated using a one-end correction version of the equation by Franks and Farquhar (2001):


$$g_{\mathrm{sw}.\max}=\frac{d\times \mathrm{SD}\times PA_{\max}}{v\times \left(l+\frac{\pi }{2}\;\sqrt{\frac{PA_{\max}}{\pi }\;}\right)}$$

where *d* (0.0000249 m^2^ s^−1^) is the diffusivity of water in air and *v* (0.0245 m^3^ mol^−1^) is the molar volume of air in 25 °C and *π* is the mathematical constant, approximately 3.1415. PA_max_ maximum pore area, *l* (pore depth; μm) was calculated as half of the GCW and SD is SD. *g*_sw.max_ from adaxial leaf surface (*g*_ad_) and abaxial leaf surface (*g*_ab_) were calculated separately. Then, total leaf anatomical *g*_sw.max_ (*g*_total_) for each accession was calculated as the sum of *g*_ad_ and *g*_ab_.

#### Leaf physiological (gas exchange) trait measurements

Following the completion of stomatal sampling, multiple physiological or gas exchange parameters on the leaf level were recorded using the LI-6800 Portable Photosynthesis system (LI-COR, Lincoln, NE, USA) (Fig. S14b). For each measurement, we used a 6 cm^2^ standard leaf chamber. Measurements were performed on a clear sunny day under natural PPFD of 1400–1,600 µmol m^−2^ s^−1^ and ambient O_2_ condition (approximately 21%) between 10.00 AM and 12.30 PM. The environment inside the leaf chamber was maintained to match plant growth conditions, which were 28–30 °C leaf temperature, 1.5–2.0 vapor pressure deficit (VPD), constant airflow (600 µmol s^−1^), and 60 ± 5% RH. The ambient atmospheric CO_2_ concentration (C_a_) was set at 410 µmol mol^−1^ which was supplied through an external CO_2_ cylinder. Net photosynthetic rate (*A*_n_), intercellular CO_2_ concentration (C_i_), stomatal gas conductance to water (g_sw_), and intrinsic water-use efficiency (*_i_*WUE; the ratio of A_n_ to ^g^_sw_) were recorded when *A*_n_, *g*_sw_, and C*_i_* became stable.

#### Leaf functional trait measurements

Following the completion of leaf physiological measurements, leaf functional traits including LW (cm), LT (mm), and total CC (SPAD units) were measured in 3 biological replications per accession. Briefly, LT and LW were measured using a digital thickness gauge micrometer with 0.001 mm resolution and a ruler, respectively (Fig. S14c). CC was estimated nondestructively using MC-100 chlorophyll concentration meter (Apogee, Logan, UT, USA). LMA (g m^−2^) was calculated as the ratio between leaf dry mass (g) and leaf area (m^2^). In brief, 10 leaf discs of 1.69 cm^2^ each were collected, oven-dried to a constant weight at about 80 °C for 2 days, and their DW was measured.

### Linear mixed model for leaf anatomical, physiological, and functional traits

A linear mixed-effects model was applied to assess the influence of both fixed and random effects on leaf anatomical, physiological, and functional traits. Because sorghum accessions were planted across multiple rows and columns in the experimental field, spatial effects were accounted for by including row and column as fixed effects. Additionally, average daily temperature (AveT) and average humidity (AveH) on the day of phenotyping (across batches) were incorporated as fixed effects to control for environmental variation. Accession was modeled as a random effect to capture genetic variation among sorghum accessions. Trait means were modeled using the restricted maximum likelihood method implemented via the lmer() function from the lme4 package in R (Bates et al. 2015), with the following model structure:


$$\begin{array}{rl} \mathrm{model} & =\mathrm{lmer}(\mathrm{trait}\;\mathrm{of}\;\mathrm{intrest}∼\mathrm{row}+\mathrm{column}\\ & \;+\;\mathrm{AveT}+\mathrm{AveH}+(1+\mathrm{accession}),\mathrm{data}=\mathrm{my}\_\mathrm{data}) \end{array}$$

Model selection was guided by the Akaike Information Criterion, comparing alternative models that included or excluded AveT and AveH. Predicted values for each response variable were obtained using the predict() function in R, generating both fixed-effect estimates and random-effect predictions. These predicted values were used in subsequent data visualization, correlation analyses, and GWASs.

### GWAS analysis and colocalized gene annotation

GWAS were conducted using 569,305 SNPs previously reported by Miao et al. (2020), aligned to the *Sorghum bicolor* BTx623 reference genome version 3.1.1 (McCormick et al. 2018). Missing genotype data were imputed following the pipeline described by Mural et al. (2021). SNPs with a minor allele frequency below 3% were excluded, resulting in a filtered dataset of 234,264 SNPs across 358 sorghum accessions. GWAS were performed for a range of phenotypic traits, including stomatal traits (SD_total_, SPALA_total_), physiological traits (*A*_n_, *g*_sw_, *_i_*WUE), and functional traits (LW, LT, LMA, CC). Association analyses were carried out using the Fixed and Random Model with Circulating Probability Unification (FarmCPU) algorithm, implemented in the “rMVP” R package (Liu et al. 2016; Yin et al. 2021). To account for population structure, the first 3 principal components (PCs) were included as covariates, and a kinship matrix was incorporated as a random effect, calculated internally by the FarmCPU model (Mural et al. 2021).

To control for false positive, a Bonferroni multiple testing correction threshold of 2.13 × 10^−7^ (α/number of markers, where α = 0.05) was applied. To examine the degree of model fitness, quantile–quantile (Q–Q) plots of each GWAS model were constructed via plotting quantile distribution of observed −log_10_(*P*) on the y-axis versus the quantile distribution of expected −log_10_(*P*) on *x*-axis (Fig. S4). Multi-trait Manhattan plots were created using the “CMplot” R package (v3.6.2) (Yin 2024). To identify candidate genes, genomic region 75 kb up- and downstream from significant SNPs were scanned for annotated genes using the *Sorghum bicolor* reference genome v3.1.1, considering a linkage disequilibrium decay range of 150–600 kb (Ortiz et al. 2022; Singh et al. 2025). Haplotype analysis of selected SNPs was performed based on reference and alternative allele combinations, and their distribution and effects were examined across sorghum accessions.

### Growth chamber experiment

Eight sorghum accessions representing distinct haplotype combinations (PI 533985, PI 656080, PI 607931, PI 656092, PI 656028, PI 576348, PI 656018, PI 576350) were selected based on significant variability in traits such as SD_total_, *A*_n_, *g*_sw_, and _i_WUE under natural field conditions. To validate the effect of haplotype variation on stomatal sensitivity and water-use efficiency in different environments, a growth chamber experiment was performed under 2 water regimes: WW and WS. Plants were grown in 7.5-L plastic pots (with a dimension of 22.8 cm in height, 24.1 cm in width at the top, and 19.0 cm in width at the bottom), filled with a commercial peat-and-perlite potting mix (Berger 6, Berger, Canada). For logistical efficiency, the experiment was divided into 4 sowing batches (March 27, April 1, April 6, and 11 April 2023), with PI 564163 used as a control (check) in each batch. Each accession was represented by 4 pots per batch, resulting in a total of 128 pots (8 accessions × 4 pots × 4 batches).

Growth conditions were maintained in a walk-in growth chamber (Environmental Growth Chamber, Chagrin Falls, OH) at 60% RH, with a temperature regime of 28 °C during the day and 24 °C at night. The photoperiod was set to 16 hours of light (7:00 AM–11:00 PM) and 8 hours of darkness (11:00 PM–7:00 AM), with a constant light intensity of 1,600 μmol m⁻^2^ s⁻^1^ PPFD. Three seeds were sown per pot at a depth of 2 cm, and after germination, seedlings were thinned to retain 2 healthy plants per pot. All plants were initially WW and fertilized as needed to ensure optimal growth. Upon reaching the 5-leaf stage (approximately 25–28 days post-germination), pots were divided equally into 2 treatment groups. Half of the pots (2 per accession) continued under WW conditions, while the other half were subjected to a 3-day dry-down period to induce WS. Following treatment imposition, both WW and WS plants were used for chlorophyll fluorescence and gas exchange measurements under variable light intensities, as described in subsequent sections.

### Chlorophyll fluorescence measurements

Chlorophyll fluorescence was performed using a 6 cm^2^ standard leaf chamber of LI-6800-01A fluorometer (LI-COR, Lincoln, NE, USA) on the fully expanded sixth leaf from WW and WS plants. These plants were in complete darkness overnight, and fluorescence measurements started just 1 h before dawn. The leaf temperature was set at 24 °C, light intensity at 0 μmol m^−2^ s^−1^, CO_2_ concentration at 410 μmol mol^−1^, and RH at 60%, as described above. Minimum chlorophyll fluorescence (*F_0_*) was determined by a weak, measuring beam, while maximum chlorophyll fluorescence (*F_m_*) level was achieved upon the application of a saturating flash of light (8,000 μmol photons m^−2^ s^−1^) for 1 s in the dark-adapted state. Variable chlorophyll fluorescence (*F*_v_) was calculated as *F*_m—_*F_0_*, and maximum quantum yield of PSII as *F*_v_/*F*_m_.

### *A*_n_ -response curve under varied light conditions

Following fluorescence measurements and 2 h of light exposure, light response curves of photosynthesis were conducted between 8.30 AM and 3.30 PM. Measurements were performed using a 6 cm^2^ standard leaf chamber of LI-6800-01A fluorometer (LI-COR, Lincoln, NE, USA) on the fully expanded sixth leaf of plants subjected to WW and WS treatments. Leaves were clamped into the chamber under controlled environmental conditions: leaf temperature was maintained at 28 °C, CO_2_ concentration was set at 410 μmol mol^−1^ (supplied via a CO_2_ cylinder to simulate the current atmospheric CO_2_ level), and RH was initially set at 60%, yielding a VPD of approximately 2 kPa. Before initiating the light response protocol, leaves were initially acclimatized at the highest PPFD of 1,600 μmol m^−2^ s^−1^ by a red-blue light-emitting diode light source for at least 20 min. Once *g*_sw_ attained steady state rates, the PPFD was sequentially lowered in a stepwise manner through the following levels:1,500, 1,400, 1,200, 1,000, 800, 600, 400, 200, 100, 50 μmol m^−2^ s^−1^. At each PPFD level, *g*_sw_ was allowed to reach a steady state before measurements were recorded, and the next stepwise change was initiated. Each full light response curve required approximately 80–90 min to complete. A total of 48 leaves were measured, encompassing 8 sorghum accessions, with 3 biological replicates per accession under 2 water regimes (WW and WS).

### Fitting Ball–Woodrow–Berry model under varied light conditions

The BWB model is a semi-empirical model based on the relationship between stomatal gas conductance and photosynthetic rate. Ball et al. (1987) established a relationship between *g*_sw_, *A*_n_, CO_2_ concentration at leaf surface (*C*_s_), and RH (*H*_s_) as


$$g_{sw}=m\frac{A_{n}\times H_{s}}{C_{s}}+g_{o}$$

where *g_o_* is the residual stomatal conductance when *A_n_* approaches zero, *m* is the slope of the relationship between *g*_sw_ and *A*_n_ × *H*_s_/*C*_s_ (the ball-index), which is also called the stomatal sensitivity factor (Harley and Tenhunen 1991).

### Statistical data analyses and visualization

Most statistical analyses and data visualizations were performed using RStudio (version 4.3.3; R Core Team 2024). Phenotypic trait distributions were visualized using histograms, and descriptive statistics, including the coefficient of variation and skewness, were calculated to assess variability. To investigate the multivariate structure and clustering of phenotyped traits, PCA was performed using the PCA() function from the “FactoMineR” package. Trait relationships were further examined through linear regression using the lm() function, and pairwise Pearson correlation coefficients (*r*) with associated *P*-values were computed and visualized using the “ggcorrplot” package. Additional graphical representations were created using Microsoft Excel. Group differences were assessed using Student's *t*-tests (*P* < 0.05 = *, *P* < 0.01 = **, *P* < 0.001 = ***, ns = not significant). For comparisons involving 1, 2, or more independent variables, 1-way and 2-way analysis of variance (ANOVA) were performed using the aov() function in R. In 1-way ANOVA, the significance difference among one variable was determined by a post hoc Tukey's test. Groups with different letters in the graphs indicated significant differences among the groups at a *P* < 0.05 level of significance.

## Supplementary Material

kiag064_Supplementary_Data

## Data Availability

All datasets will be available upon request.
