# Supplementary material for: Genome-wide association studies and modeling of stomatal gas conductance reveal genetic control of water-use efficiency in sorghum
Source: Plant Physiol. 2026 Feb 16;200(3):kiag064. doi: 10.1093/plphys/kiag064 (PMC13016993; doi:10.1093/plphys/kiag064)
Supplement: kiag064_Supplementary_Data [file kiag064_supplementary_data.zip › 3_PPhys_Supplementary file_Jan 20.pdf]

## Supplementary Data

**Genome-wide association studies and modeling of stomatal gas conductance reveal genetic control of water-use efficiency in sorghum**

**Running head: GWAS and Modeling of $g_{sw}$ in Sorghum**

### Abbreviations used in this file were as follows:

- Stomata density (*SD*)
- Total stomata density ($SD_{total}$)
- Stomata length (*SL*)
- Stomatal complex width (*SCW*)
- Stomatal size (*SS*)
- Pore length (*PL*)
- Guard cell width (*GCW*)
- Maximum pore area ($PA_{max}$)
- Stomatal pore area per leaf area (*SPALA*)
- Total stomatal pore area per leaf area ($SPALA_{total}$)
- Anatomical maximum stomatal gas conductance to water vapor ($g_{sw.max}$)
- Net photosynthetic rate ($A_n$)
- Intercellular $CO_2$ concentration ($C_i$)
- Stomatal gas conductance to water vapor ($g_{sw}$)
- Intrinsic water-use efficiency ($_iWUE$)
- Leaf width (*LW*)
- Leaf thickness (*LT*)
- Leaf mass area (*LMA*)
- Chlorophyll content (*CC*)
- Efficiency of quantum yield ($F_v/F_m$)
- Photosynthetic Photon Flux Density (*PPFD*)

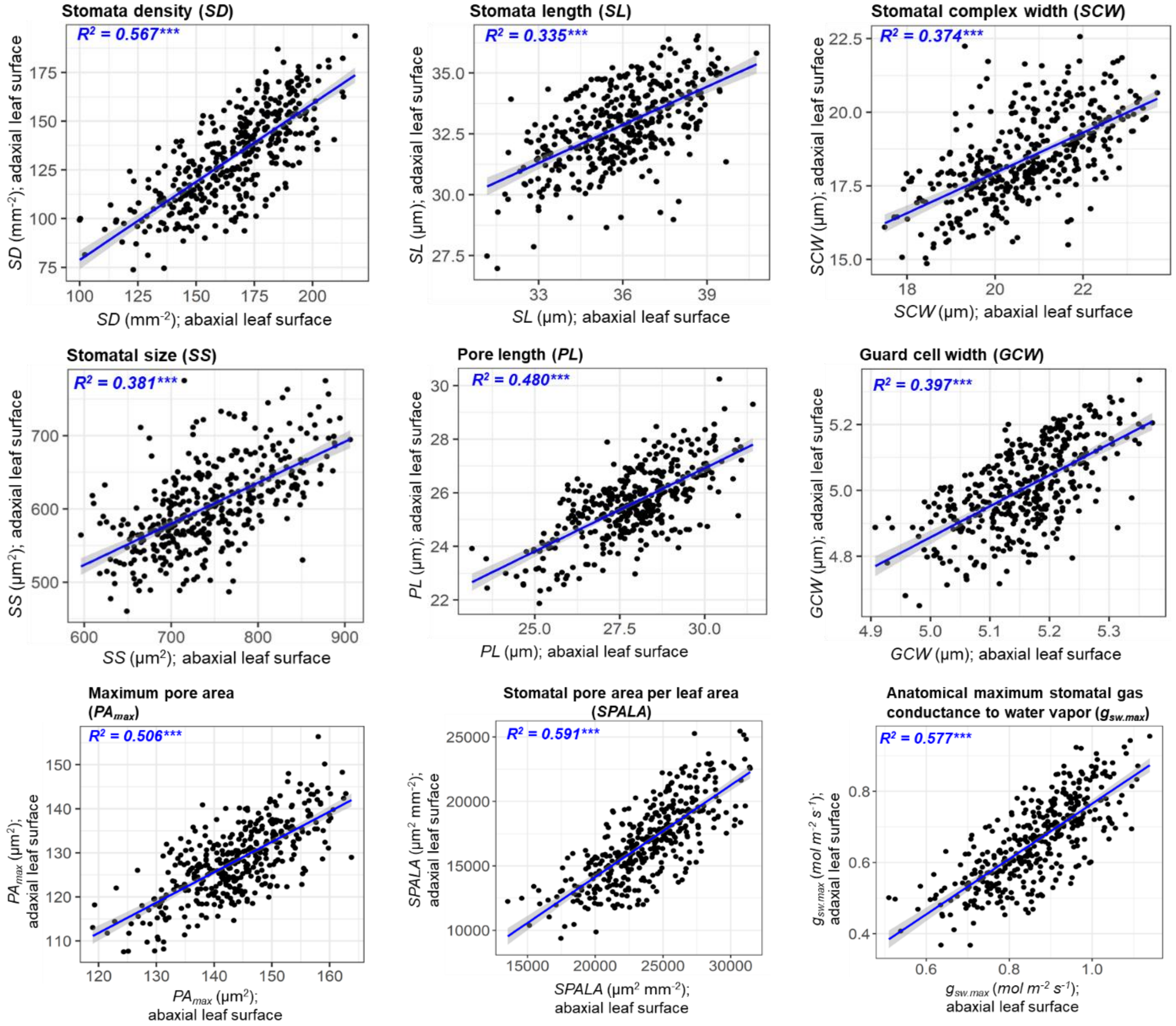


**Supplementary Figure S1. Regression analysis of leaf anatomical traits (stomatal features) on both abaxial (lower) and adaxial (upper) leaf surfaces.** The blue line represents the line of best fit, and $R^2$ indicates the coefficient of determination. Asterisks (*, **, ***) denote significance levels of $P < 0.05$, $P < 0.01$, and $P < 0.001$, respectively. Shaded regions represent the 95% confidence intervals around fitted relationships.

| Correlation coefficient (r) | | | | |
|---|---|---|---|---|
| -1 | -0.5 | 0 | 0.5 | 1 |

| | $A_n$ | $C_i$ | $g_{sw}$ | $_iWUE$ |
|---|---|---|---|---|
| $A_n$ | 1.0 | | | |
| $C_i$ | 0.870 | 1.0 | | |
| $g_{sw}$ | 0.699 | 0.618 | 1.0 | |
| $_iWUE$ | -0.174 | -0.151 | -0.802 | 1.0 |

**Supplementary Figure S2. Correlation analyses among leaf physiological (gas exchange) traits.** Statistically significant correlations ($P < 0.05$) are shown as colored cells, with red indicating positive correlations and blue indicating negative correlations. Color intensity reflects the strength of the correlation coefficient ($r$, ranging from -1 to 1). Non-significant correlations are denoted by '×'. **Abbreviations:** $A_n$: net pho Net photosynthetic rate, $C_i$: Intercellular $CO_2$ concentration, $g_{sw}$: Stomatal gas conductance to water vapor, $_iWUE$: Intrinsic water-use efficiency.

| Correlation coefficient (*r*) | | | | |
|---|---|---|---|---|
| -1 | -0.5 | 0 | 0.5 | 1 |

| | ***LW*** | ***LT*** | ***LMA*** | ***CC*** |
|---|---|---|---|---|
| ***LW*** | 1.0 | | | |
| ***LT*** | 0.440 | 1.0 | | |
| ***LMA*** | 0.354 | 0.803 | 1.0 | |
| ***CC*** | 0.416 | 0.679 | 0.561 | 1.0 |

**Supplementary Figure S3. Correlation analysis among leaf functional traits.** Statistically significant correlations ($P < 0.05$) are shown as colored cells, with red indicating positive correlations and blue indicating negative correlations. Color intensity reflects the strength of the correlation coefficient ($r$, ranging from -1 to 1). Non-significant correlations are denoted by '×'. **Abbreviations:** *LW*: Leaf width, *LT*: Leaf thickness, *LMA*: Leaf mass area, *CC*: Chlorophyll content.

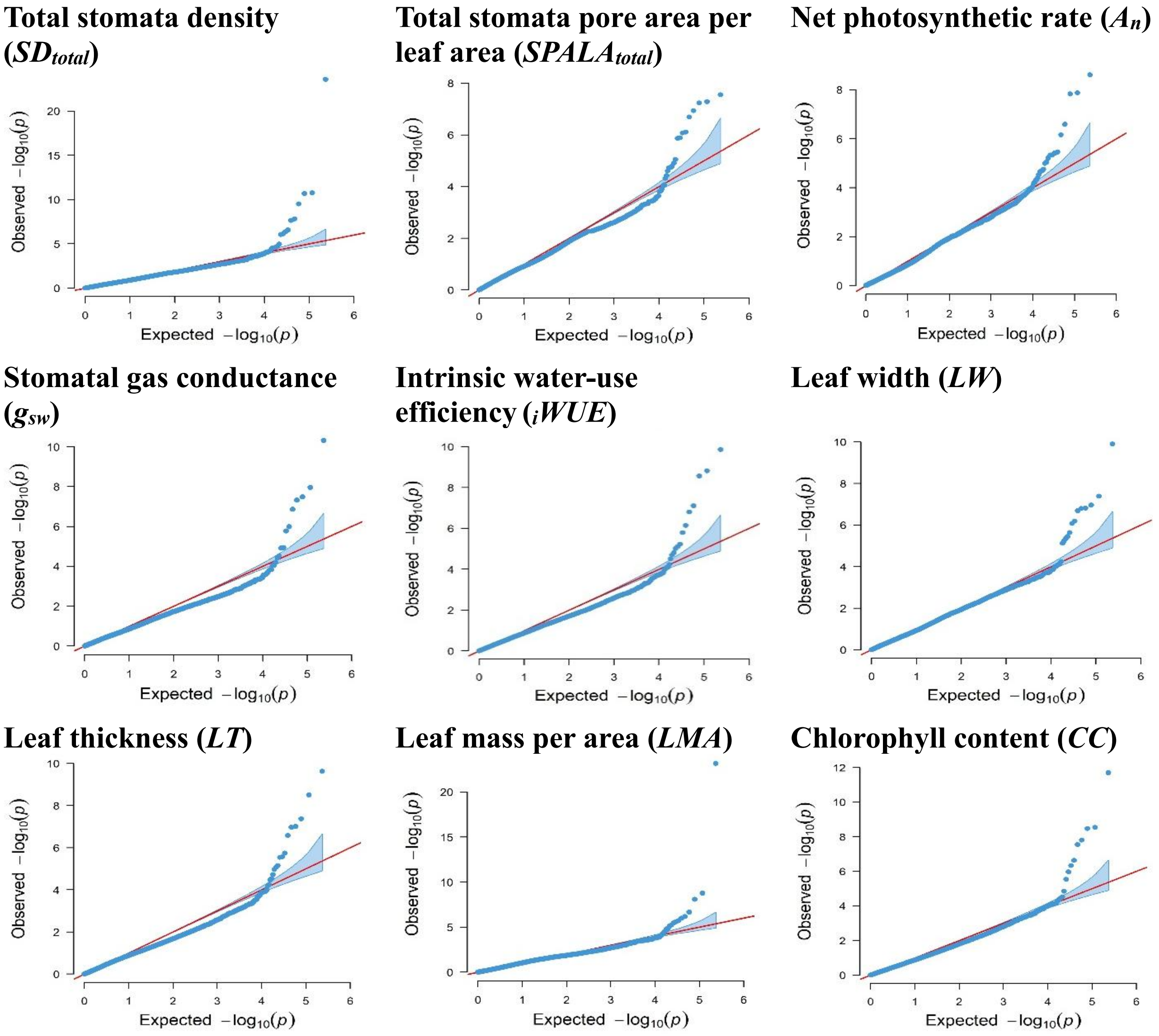


**Supplementary Figure S4. Quantile–quantile (Q-Q) plots showing the results of the genome-wide association studies (GWAS) for leaf anatomical, physiological, and functional traits.** GWAS analyses were conducted using the FarmCPU model on the Sorghum Association Panel. The x-axis represents the expected -$\log_{10}(P)$ values under the null hypothesis, the y-axis displays the observed -$\log_{10}(P)$ values. Deviations from the expected line, particularly in the upper tail, indicate significant marker-trait associations. Shaded regions denote the 95% confidence intervals around the expected *P*-values.

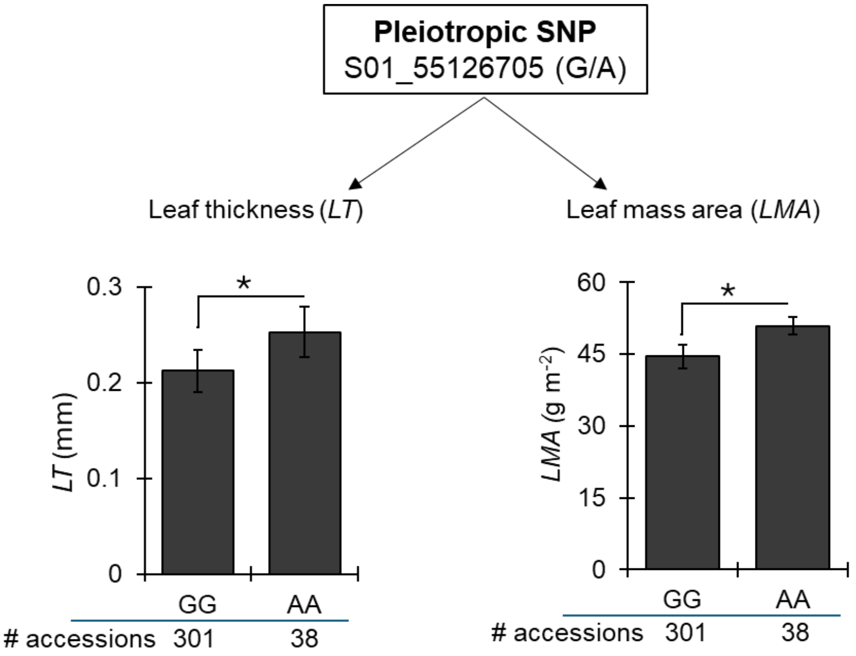


**Supplementary Figure S5. Allelic variation of a pleiotropic SNP (S01_55126705) associated with leaf thickness (*LT*) and leaf mass area (*LMA*).** Values represent means ± standard deviation (SD) for sorghum accessions carrying different alleles. Statistical significance between allele groups was assessed using Student's t-test (*P < 0.05*, P < 0.01**, P < 0.001***;* ns = not significant).

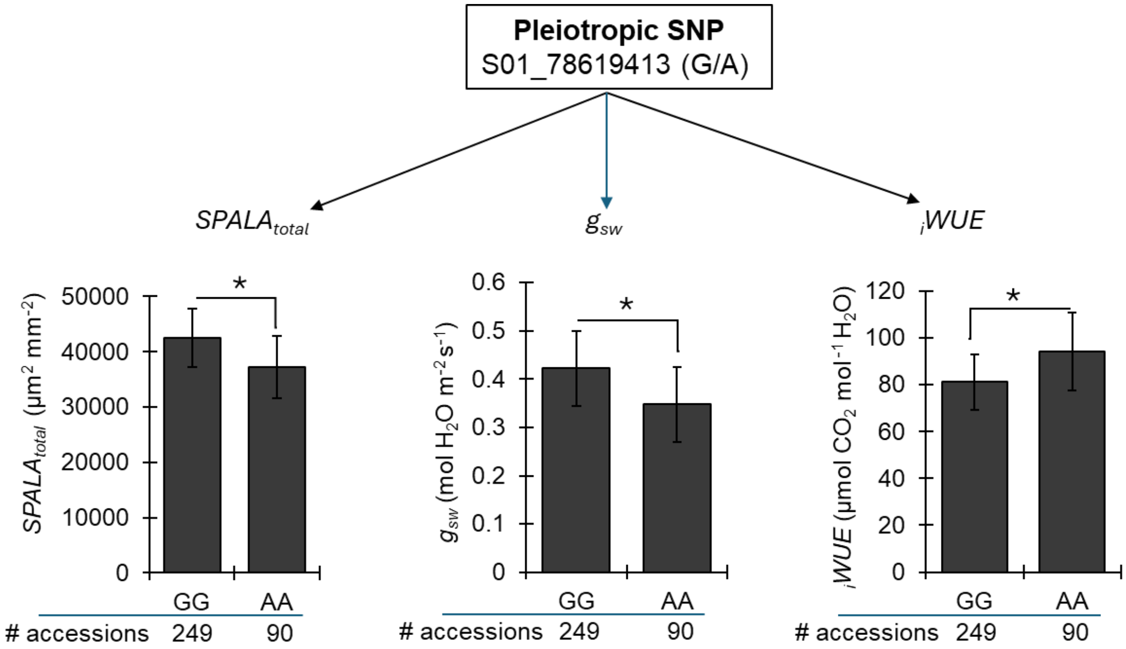


**Supplementary Figure S6. Allelic variation of a pleiotropic SNP (S01_78619413) associated with total stomatal pore area per leaf area (*$SPALA_{total}$*), stomatal gas conductance to water vapor (*$g_{sw}$*) and intrinsic water-use efficiency (*$_iWUE$*).** Values represent means ± standard deviation (SD) for sorghum accessions carrying different alleles. Statistical significance between allele groups was assessed using Student's t-test (*$P < 0.05$*, $P < 0.01$**, $P < 0.001$***;* ns = not significant).

Correlation coefficient (*r*): -1.0 | -0.5 | 0.0 | 0.5 | 1.0

| SNP and associated traits | S01_17830866 ($SD_{total}$) | S01_49472845 ($_{i}WUE$) | S01_55126705 (*LT, LMA*) | S01_57257980 ($g_{sw}$) | S01_6079837 ($g_{sw}$) | **S01_77550396 ($SD_{total}$)** | S01_77672597 ($SPALA_{total}$) | **S01_78561058 ($A_n$)** | **S01_78619413 ($SPALA_{total}$, $g_{sw}$, $_{i}WUE$)** |
|---|---|---|---|---|---|---|---|---|---|
| S01_17830866 ($SD_{total}$) | 1.000 | | | | | | | | |
| S01_49472845 ($_{i}WUE$) | -0.183 | 1.000 | | | | | | | |
| S01_55126705 (*LT, LMA*) | -0.100 | -0.160 | 1.000 | | | | | | |
| S01_57257980 ($g_{sw}$) | -0.042 × | -0.037× | 0.285 | 1.000 | | | | | |
| S01_6079837 ($g_{sw}$) | -0.049 × | 0.093 | -0.082 | 0.008 × | 1.000 | | | | |
| **S01_77550396 ($SD_{total}$)** | 0.151 | 0.035 × | 0.026 × | -0.022 × | 0.069 × | 1.000 | | | |
| S01_77672597 ($SPALA_{total}$) | -0.025 × | -0.084 | 0.364 | 0.095 | 0.000 × | 0.462 | 1.000 | | |
| **S01_78561058 ($A_n$)** | 0.063 × | -0.119 | -0.033 × | 0.179 | 0.022 × | -0.101 | 0.011 × | 1.000 | |
| **S01_78619413 ($SPALA_{total}$, $g_{sw}$, $_{i}WUE$)** | 0.070 × | -0.077 | 0.361 | 0.322 | -0.046 × | 0.483 | 0.471 | 0.141 | 1.000 |

**Supplementary Figure S7. Correlation analysis among SNPs located on chromosome 1.** Statistically significant correlations ($P < 0.05$) are shown as colored cells, with red indicating positive correlations and blue indicating negative correlations. Color intensity reflects the strength of the correlation coefficient (*r*, ranging from -1 to 1). Non-significant correlations are denoted by '×'. **Abbreviations:** $SD_{total}$: total stomata density, $SPALA_{total}$: total stomata pore area per leaf area, *LT*: leaf thickness, *LMA*: leaf mass area, $A_n$: net photosynthetic rate, $g_{sw}$: stomatal gas conductance to water vapor, $_{i}WUE$: intrinsic water-use efficiency.

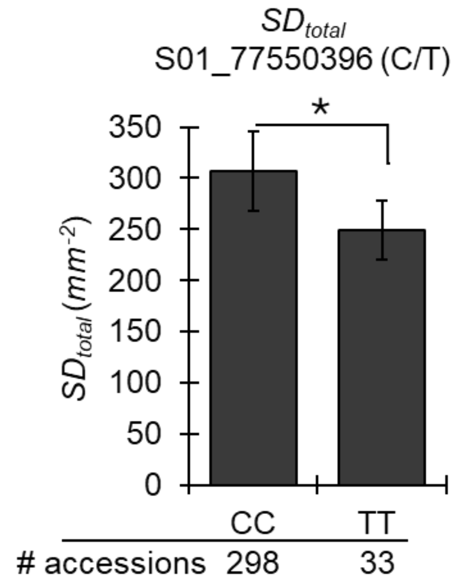


**Supplementary Figure S8. Allelic variation of SNP (S01_77550396) associated with total stomata density (*$SD_{total}$*).** Values represent means ± standard deviation (SD) for sorghum accessions carrying different alleles. Statistical significance between allele groups was assessed using Student's t-test (*$P < 0.05$*, $P < 0.01$**, $P < 0.001$***;* ns = not significant).

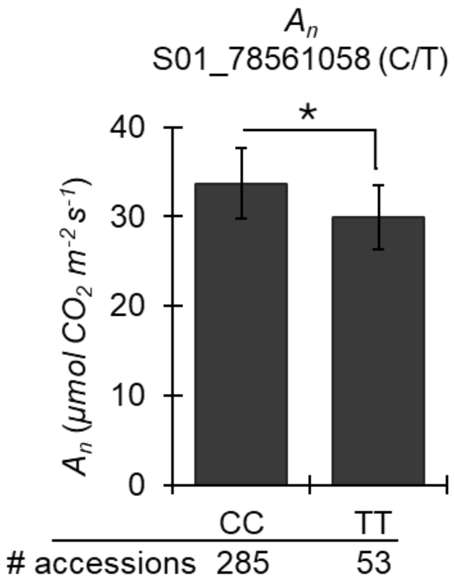


**Supplementary Figure S9. Allelic variation of SNP (S01_78561058) associated with net photosynthetic rate ($A_n$).** Values represent means ± standard deviation (SD) for sorghum accessions carrying different alleles. Statistical significance between allele groups was assessed using Student's t-test (*P < 0.05*, P < 0.01**, P < 0.001***;* ns = not significant).

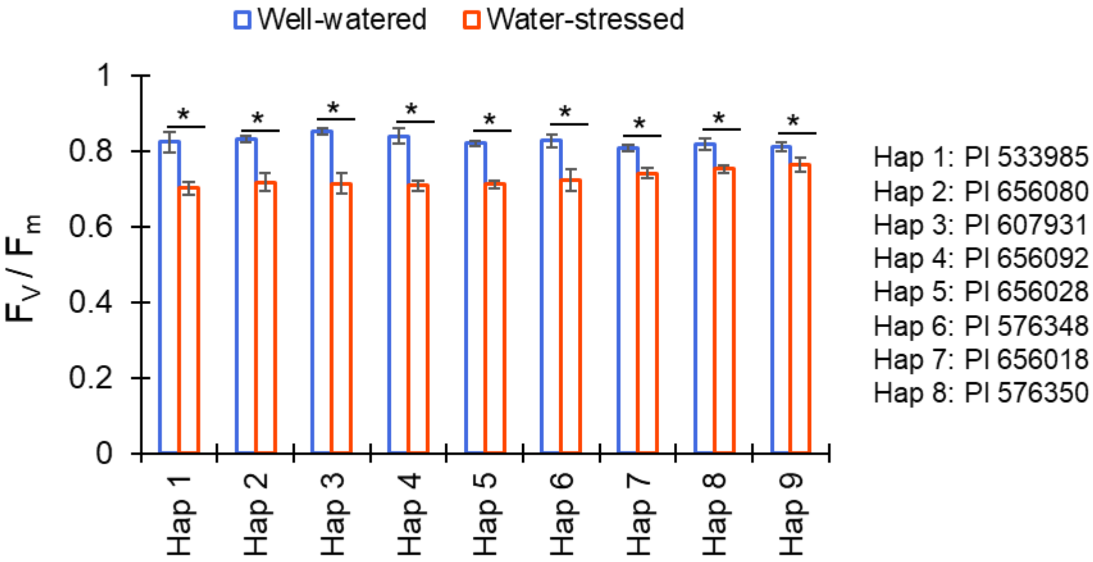


**Supplementary Figure S10. Maximum quantum yield of photosystem II ($F_v/F_m$) in dark-adapted sorghum plants grown under well-watered (WW) and water-stressed (WS) conditions.** Measurements were taken from fully expanded sixth leaves of selected sorghum accessions. Values represent means ± standard deviation (SD) from three biological replicates per accession. Statistical differences between WW and WS conditions were determined using Student's t-test (*P < 0.05*, P < 0.01**, P < 0.001****; ns = not significant).

**Net photosynthetic rate ($A_n$) in response to varying PPFD**

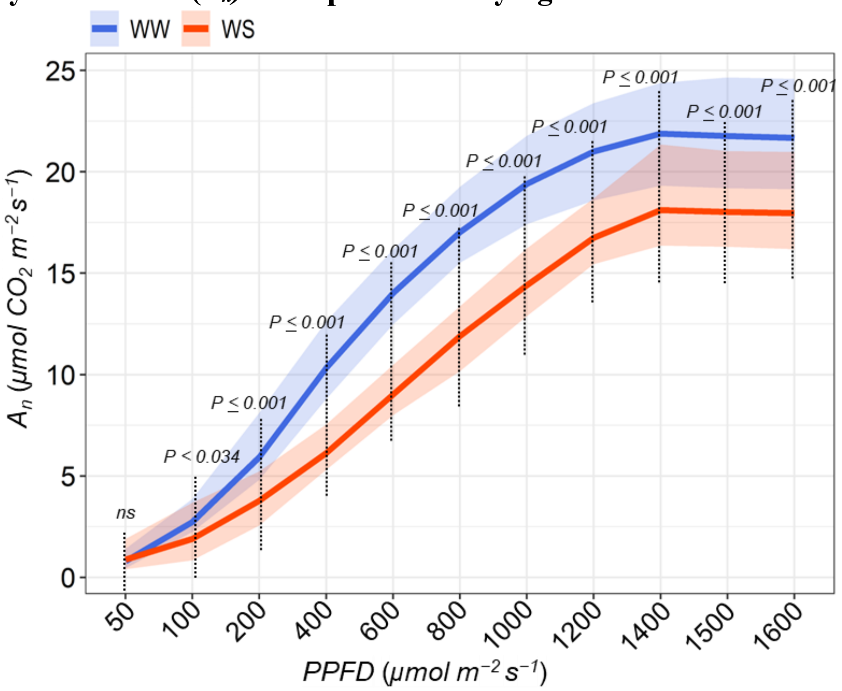


**Stomatal gas conductance to water vapor ($g_{sw}$) in response to varying PPFD**

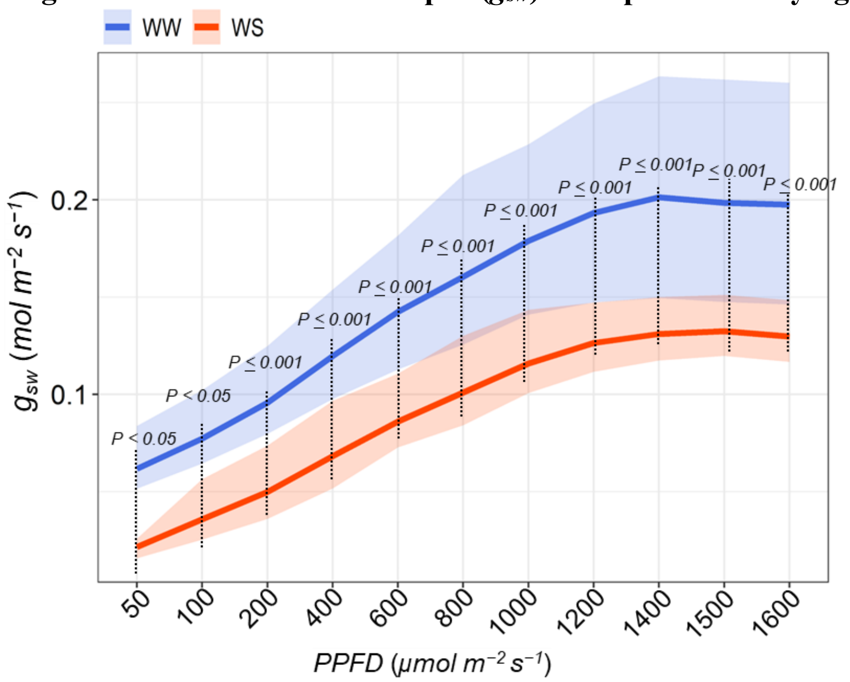


**Supplementary Figure S11. Net photosynthetic rate ($A_n$) and stomatal gas conductance to water vapor ($g_{sw}$) in response to varying Photosynthetic Photon Flux Density (PPFD).** Data was collected from fully expanded sixth leaves of sorghum plants grown under well-watered (WW) and water-stressed (WS) conditions. Solid lines represent the mean values, and shaded regions indicate ± standard deviation across 24 data points per light intensity (8 accessions × 3 biological replicates). Statistical significance between WW and WS treatments was determined using Student's *t*-test ($P < 0.05$, *$P < 0.01$, **$P < 0.001$; ns, not significant).

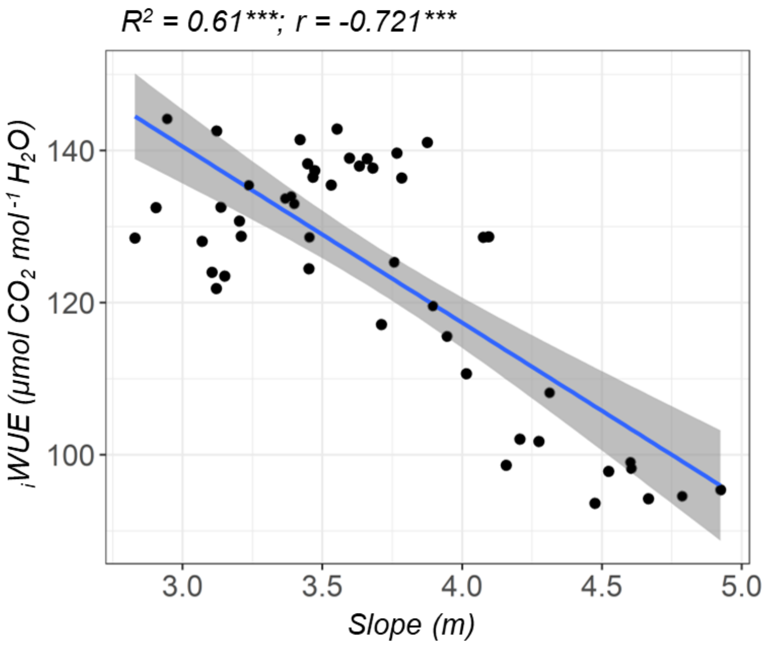


**Supplementary Figure S12. Regression analysis between intrinsic water-use efficiency (*$_i$WUE*) and slope (*m*) obtained from selected sorghum accessions grown under well-watered (WW) and water-stressed (WS) conditions.** The plot shows the line of best fit (blue line), the correlation coefficient (R), and the coefficient of determination ($R^2$). Asterisks (*, **, ***) indicate significance levels of $P < 0.05$, $P < 0.01$, and $P < 0.001$, respectively. Shaded regions represent the 95% confidence intervals around the fitted regression line.

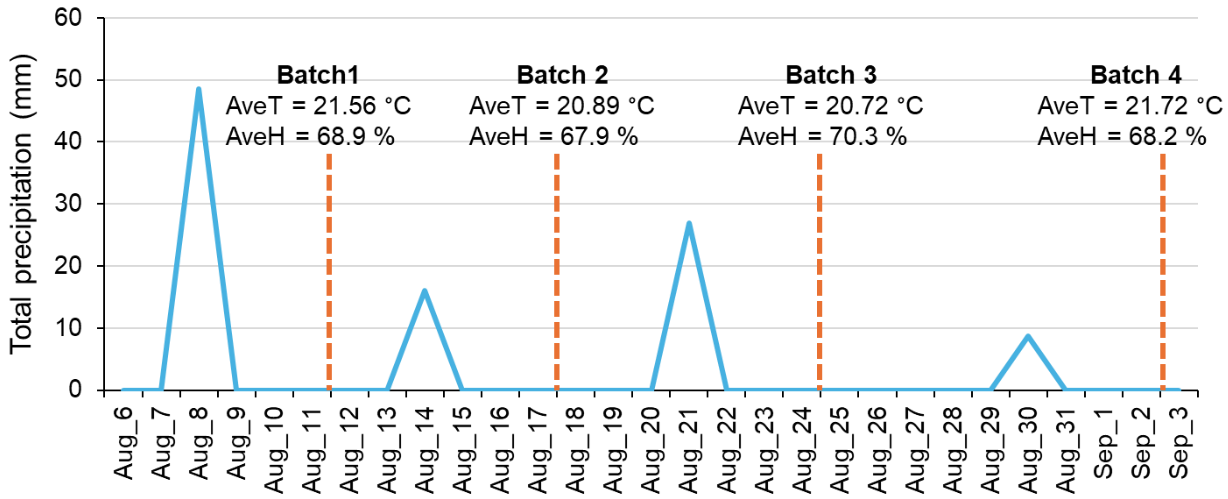


**Supplementary Figure S13. Total precipitation (in millimeters, mm) measured in August and the early week of September 2022 at the Michigan State University Research Farm.** The orange dotted line indicates different batches with average daily temperature (AveT) and average humidity (AveH) on which leaf anatomical, physiological, and functional data were collected. The data was obtained from the Weather Underground site. https://www.wunderground.com/weather/us/mi/meridian-charter-township/KMIMERID11.

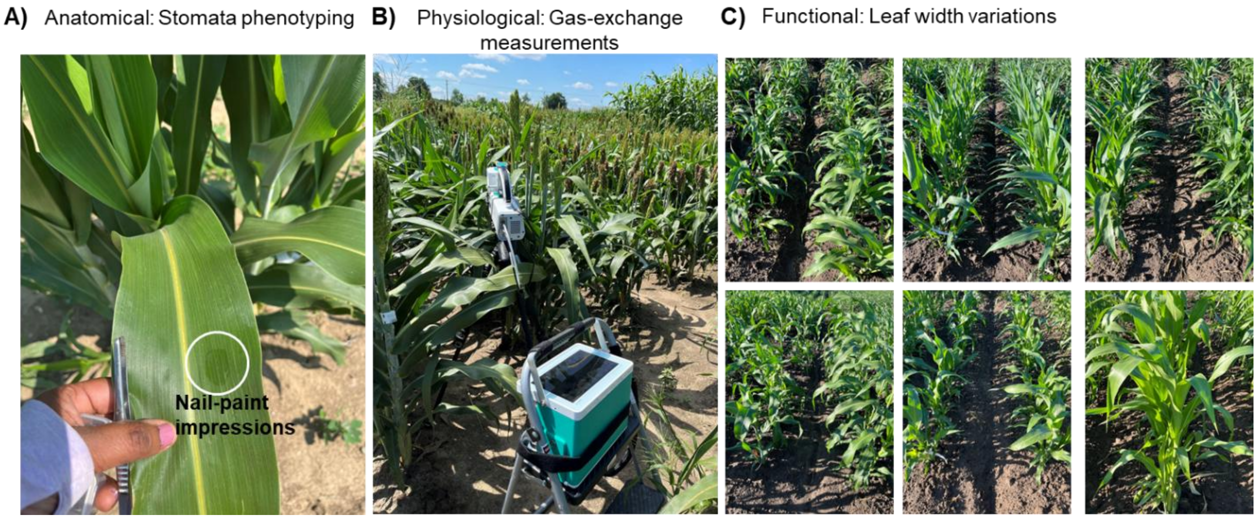


**Supplementary Figure S14. Phenotyping of leaf anatomical, physiological, and functional traits under natural field conditions.** Anatomical traits were assessed by collecting nail polish impressions of the leaf surface to evaluate stomatal characteristics. Physiological traits, including photosynthetic parameters, were measured using the LI-6800 Portable Photosynthesis System. Functional traits, such as leaf width variation, were recorded using a ruler.
